# Polycomb recruitment attenuates retinoic acid–induced transcription of the bivalent *NR2F1* gene

**DOI:** 10.1093/nar/gkt367

**Published:** 2013-05-10

**Authors:** Kristian B. Laursen, Nigel P. Mongan, Yong Zhuang, Mary M. Ng, Yannick D. Benoit, Lorraine J. Gudas

**Affiliations:** Department of Pharmacology, Weill Cornell Medical College of Cornell University, 1300 York Avenue, New York, NY 10065, USA

## Abstract

Polycomb proteins play key roles in mediating epigenetic modifications that occur during cell differentiation. The Polycomb repressive complex 2 (PRC2) mediates the tri-methylation of histone H3 lysine 27 (H3K27me3). In this study, we identify a distinguishing feature of two classes of PRC2 target genes, represented by the *Nr2F1* (*Coup-TF1*) and the *Hoxa5* gene, respectively. Both genes are transcriptionally activated by all-*trans* retinoic acid (RA) and display increased levels of the permissive H3K9/K14ac and tri-methylated histone H3 lysine 4 epigenetic marks in response to RA. However, while in response to RA the PRC2 and H3K27me3 marks are greatly decreased at the *Hoxa5* promoter, these marks are initially increased at the *Nr2F1* promoter. Functional depletion of the essential PRC2 protein Suz12 by short hairpin RNA (shRNA) technology enhanced the RA-associated transcription of *Nr2F1*, *Nr2F2*, *Meis1*, *Sox9* and *BMP2*, but had no effect on the *Hoxa5*, *Hoxa1*, *Cyp26a1*, *Cyp26b1* and *RARβ_2_* transcript levels in wild-type embryonic stem cells. We propose that PRC2 recruitment attenuates the RA-associated transcriptional activation of a subset of genes. Such a mechanism would permit the fine-tuning of transcriptional networks during differentiation.

## INTRODUCTION

The ability to self-renew and differentiate into specific cell lineages in response to external stimuli is a unique property of pluripotent stem cells. This ability makes embryonic stem (ES) cells an excellent model for *in vivo* differentiation (1). All-*trans* retinoic acid (RA), a metabolite of vitamin A, induces epigenetic and transcriptional changes underpinning the differentiation of various stem cells, including ES cells (2,3). Several key regulators of stem cell differentiation exhibit a bivalent chromatin structure possessing both repressive and permissive histone modification, tri-methylated histone H3 lysine 27 (H3K27me3) and tri-methylated histone H3 lysine 4 (H3K4me3), respectively (4). Differentiation of stem cells, e.g. during neurogenesis, involves epigenetic changes, which resolves bivalent regions into either active H3K4me3-rich, or repressive H3K27me3-rich, domains (5,6).

The Polycomb Repressive Complex 2 (PRC2) is a multi-protein complex that confers transcriptional repression via the placement of the repressive H3K27me3 histone mark. Indeed, Polycomb repressive complexes (PRC1/2) silence many genes in ES cells (7,8). The PRC2 protein Ezh2 is a H3K27-specific histone methyltransferase that, via epigenetic modification of histones, controls aspects of cell fate choice during differentiation (9). Ezh2 deposits the H3K27me3 repressive mark recognized by PRC1 factors, which leads to Ring1 mono-ubiquitination of histone H2A lysine 119 (8,10,11). Ezh2, Suz12 and Eed proteins form the core of the PRC2 complex, and the methyltransferase activity of PRC2 requires both Ezh2 and Suz12 (12,13). While the role of epigenetic modifications at promoter proximal regions has been extensively studied (4), the mechanisms by which epigenetic changes at distal enhancer sites influence transcription and how these relate to the PRC function at proximal promoters are only now emerging (14,15).

Vitamin A (retinol) and its natural and synthetic analogs, retinoids, exert profound effects on many biological processes [for review see (2,3)]. The retinol metabolite all-*trans* RA mediates most biological effects of retinol (16), and has been implicated in numerous *in vivo* differentiation pathways (17). The actions of RA are primarily mediated by two classes of nuclear retinoid receptors: retinoic acid receptors (RARs) and retinoid X receptors (RXRs) (18). These nuclear receptors are members of the steroid hormone or nuclear receptor superfamily that also includes estrogen, androgen, thyroid hormone, peroxisome proliferator activated receptors and vitamin D receptors. These receptors act as ligand-modulated transcription factors that activate transcription of specific target genes (19,20). We have previously shown that RA treatment of ES and F9 cells leads to the removal of the PRC2 complex from several RA target genes, including *Hoxa1*, *Cyp26a1* and *RARβ_2_* (21–24), and that the removal of PRC2 is a key step in the transcriptional induction of these direct/primary RA target genes (21,25). It is unclear whether PRC2 displacement is a common feature associated with RA-induced transcription.

*Nr2F1* and *Nr2F2* are also referred to as the chicken-ovalbumin upstream promoter-transcription factors (Coup-TF1/2). Nr2F1 and Nr2F2 belong to a diverse group of nuclear receptors, which are termed orphan nuclear receptors because physiological ligands have not yet been identified (26). In mammals, only two genes, *Nr2F1* (*Coup-TF1*, *EAR-3*) and *Nr2F2* (*Coup-TF2*, *ARP-1*), have been identified, but homologs have been cloned from numerous species (27,28). The high degree of evolutionary conservation of the Coup-TF proteins strongly suggests that they are primordial members of the nuclear receptor family and that they have important biological functions (29–32). We and others have shown that RA-induced endodermal differentiation is associated with increased *Nr2F1* mRNA levels (33–35). We have further demonstrated that ectopic expression of *Nr2F1* enhances the RA-induced differentiation of ES cells into extra-embryonic endoderm (35), which may suggest that induction of *Nr2F1* is a key event in the generation of endodermal tissue. Consistent with this expression of *Laminin*, which is required to separate the primitive endoderm from the epiblast, is regulated by Nr2F1 (36). Although Nr2F2 has been shown to bind RA (37), it is not clear if *Nr2F2* is activated by RA under physiological conditions. The expression patterns of *Nr2F1* and *Nr2F2* are partially overlapping in the early mouse embryo (E7.5), but later in development, *Nr2F1* is expressed mainly in the nervous system, whereas *Nr2F2* is predominantly expressed in the mesenchyme of internal organs such as the pancreas (28). The roles of Nr2F1 and Nr2F2 in RA-induced endodermal differentiation and the potential association with RA suggest that these transcription factors are key players in mediating the cellular response to RA (38), yet how the expression of the *Nr2F1* and *Nr2F2* is regulated by RA remains largely unknown.

In this study, we first evaluate the effects of RA on the epigenetic states of *Nr2F1*, *Nr2F2*, *Hoxa5* and *Hoxa1* in F9 embryonal carcinoma stem cells. We demonstrate differential Suz12 dynamics between two types of PRC2 target genes, represented by the *Nr2F1* (*Coup-TF1*) and *Hoxa5* genes. We further evaluate both transcriptionally permissive and PRC2-associated repressive epigenetic marks. We extend these findings to ES cells, and delineate the functional role of Suz12 in the RA-induced transcription of the genes *Nr2F1*, *Nr2F2*, *Meis1*, *Sox9*, *BMP2*, *Hoxa5*, *Hoxa1*, *Cyp26a1*, *Cyp26b1* and *RARβ_2_*. Our findings identify PRC2 dynamics as a distinguishing feature between two classes of RA-inducible stem cell genes, both of which include several key regulators of differentiation. We suggest that in addition to maintaining transcriptional repression, the PRC2 complex attenuates the transcriptional activation of specific genes during stem cell differentiation.

## MATERIALS AND METHODS

### Cell culture and RNA extraction

F9 wild-type (WT), *RARα^−/−^*, *RARβ_2_^−/−^* and *RARγ^−/−^* embryonal teratocarcinoma stem cells were cultured as described (39–41). The *RAR* knockout cell lines were validated by reverse transcriptase-polymerase chain reaction (RT-PCR) (Supplementary Figure S1). WT (J1) and knockdown ES lines were cultured as described (23). Total cellular RNA was extracted using Trizol (Invitrogen, CA) according to the manufacturer's protocol.

### Generation of Suz12 knockdown cell lines

Generation of viral particles and transduction of ES cells was previously described (42,43). In brief, knockdown vectors pLKO shSuz12 (Cat. # TRCN0000038728, Sigma Aldrich, MO) or pLKO shLuc (control), together with packaging vectors pCMVΔ8.9 and pVSV-G (Cat. #631530, Clontech, CA), were transfected into HEK293T cells using Lipofectamine 2000 (Cat. # 11668019, Invitrogen, CA). On overnight recovery, the cells were replenished with fresh media and allowed to produce virus for an additional 48 h before the supernatant was harvested, filtered through 0.45 µm filters and supplemented with polybrene. F9 and ES cells were transduced with viral supernatant in a 1:1 ratio with 2× growth medium. About 16 h later, the cells were replenished with media supplemented with puromycin (0.5 µg/ml) for 10 days of propagation in the selection media. In agreement with a previous publication (44), the knockdown was validated by western blotting.

### Semi-quantitative and Quantitative RT-PCR

Total RNA (3 μg) was used to synthesize cDNA with random primers. The cDNA synthesis was performed at 42°C for 1 h in a final volume of 20 μl using qScript (Quanta, MD). PCR were performed using 2.5 × 10^−^^2^ U *Taq* DNA polymerase (Invitrogen, CA). Each cycle included 94°C for 30 s (denaturation), 58–64°C for 45 s (annealing) and 72°C for 1 min (extension). The number of cycles required for PCR amplification in the linear range was determined experimentally for each gene. For semiquantitative PCR, amplified PCR products were resolved on 2% agarose gels and visualized by staining with ethidium bromide. Primer pairs were either obtained from published articles or designed using the PrimerSelect program (DNAstar). The primer pairs were evaluated using *in silico* PCR analysis (http://genome.ucsc.edu/) to avoid pseudogenes. All primers were designed to anneal to different exons to avoid any contribution of genomic DNA to the signal. Sequences of gene-specific primers are specified in the Supplementary Table S1. All gene expression amplicons were validated by sequencing.

### Data processing and Statistical analysis of quantitative PCR

The transcript levels in the biological triplicates (*n* = 3) were normalized to *36B4* transcript levels and statistical significance was determined by *t*-test (*P* < 0.05). The chromatin immunoprecipitation (ChIP) signals in the biological triplicates (*n* = 3) were normalized to percent input and statistical significance was determined by *t*-test (*P* < 0.05). The standard error of the mean was determined for each of the data sets (plotted as error bars in the graphs), and *P*-values <0.05 between compared samples were assigned statistical significance.

### Library screening and promoter sequence analysis

To isolate the mouse *Nr2F1* promoter, a genomic library (129SVJ Mouse Genomic Library in the Lambda FIX II vector, Stratagene, CA) was screened. A PCR product containing ∼1 kb of mouse *Nr2F1* promoter sequence was used as the probe for library screening. Positive plaques were further screened three times, and phage DNA was isolated using the Wizard kit (Promega, WI). The positive clones were verified by sequencing a portion of each clone. The inserts in the positive plaques were cloned into the pGL3 firefly luciferase reporter plasmid (Clontech, CA), and assayed for RA responsiveness. This identified a 1 kb region as sufficient for RA-induced transcription. The fragment corresponded to a 1 kb region of sequence located ∼2.4 kb upstream of the P3 RefSeq transcriptional start site (TSS; genome coordinates, NCBI bld 37, MM9, chr13:78339594-78340675).

### Transient transfections and luciferase assays

F9 cells were transfected with luciferase reporter constructs containing different lengths of 5′ flanking sequences of the *Nr2F1* gene using the Lipofectamine method (Invitrogen, CA) (45). The reporter plasmid pRL-TK (Renilla luciferase-thymidine kinase) (Promega, WI) was used as control for transfection efficiency. Cells were then cultured with or without RA for another 24 or 48 h. Firefly and Renilla luciferase activities were sequentially measured using the Dual-luciferase Reporter Assay system (Promega, WI) with a luminometer.

### ChIP assays

ChIP assays were performed as previously described (22,46,47). In brief, a one-step ChIP protocol that uses formaldehyde cross-linking was employed for histone ChIP assays. For Suz12, Ring1B and polII-CTD ChIP assays, we used a two-step ChIP protocol. Cells were resuspended in variable amounts of lysis buffer thereby normalizing for differences in cell numbers between plates. ChIPs of sonicated chromatin from 5.0 × 10^6^ F9 cells were performed with 2 µg of antibody (Ab) per ChIP. Antibodies: H3K27me3 (#07-449, Millipore, MA); H3K4me3 (#07-473, Millipore, MA); H3K9/K14ac (#06-599, Millipore, MA); Suz12 (#3737S-D39F6, Millipore, MA); Ring1B (48); polII-CTD (#MMS-134R, Covance, NJ); Rabbit-IgG (#sc-2027, Santa Cruz, CA). The primer sequences and antibodies are listed in the Supplementary Table S2. Each ChIP assay was performed at least three times starting with independently propagated cells each time (*n* ≥ 3).

### Western blots

The sodium dodecyl sulphate–polyacrylamide gel electrophoresis and western blot analyses were performed as described (22,49) using primary antibodies for Suz12 (1:1000), H3K27me3 (1:5000) and H3K4me3 (1:2000), and β-actin (1:80000, MAB1501, Millipore, MA), EZH2 (1:1000, 3147-AC22, Cell Signaling, MA), Nr2F1 (1:1000, GTX114835, GeneTex, CA) and horseradish peroxidase conjugated anti-rabbit secondary Ab (1:5000, sc-2030, Santa Cruz, CA). Each Ab was diluted in PBS with 5% Blotto (Biorad, CA) and 0.1% Tween-20. The membranes were developed with Supersignal Substrate (Pierce, IL) for 5 min and exposed to HyBlot film (Denville Scientific, NJ).

### Bioinformatics analyses

The gene location, exon–intron boundaries and TSSs were specified in accordance with the NCBI Reference Sequences (50). Processed, mapped, next-generation RNA-seq, ChIP-seq and DNA methylation/hydroxymethylation data sets for undifferentiated and RA-treated mouse ES cells were downloaded from the NCBI Geo database (http://www.ncbi.nlm.nih.gov/geo/) and viewed in the integrated genome viewer (IGV 2.0) (51). Where appropriate the GALAXY implementation of the UCSC liftover tool was used to convert genome coordinates between mouse genome builds v36 (mm8) and NCBI37 (mm9) (52).

## RESULTS

### Identification and cloning of the murine *Nr2F1* (*Coup-TF1*) 5′ flanking region

**Figure 1.**
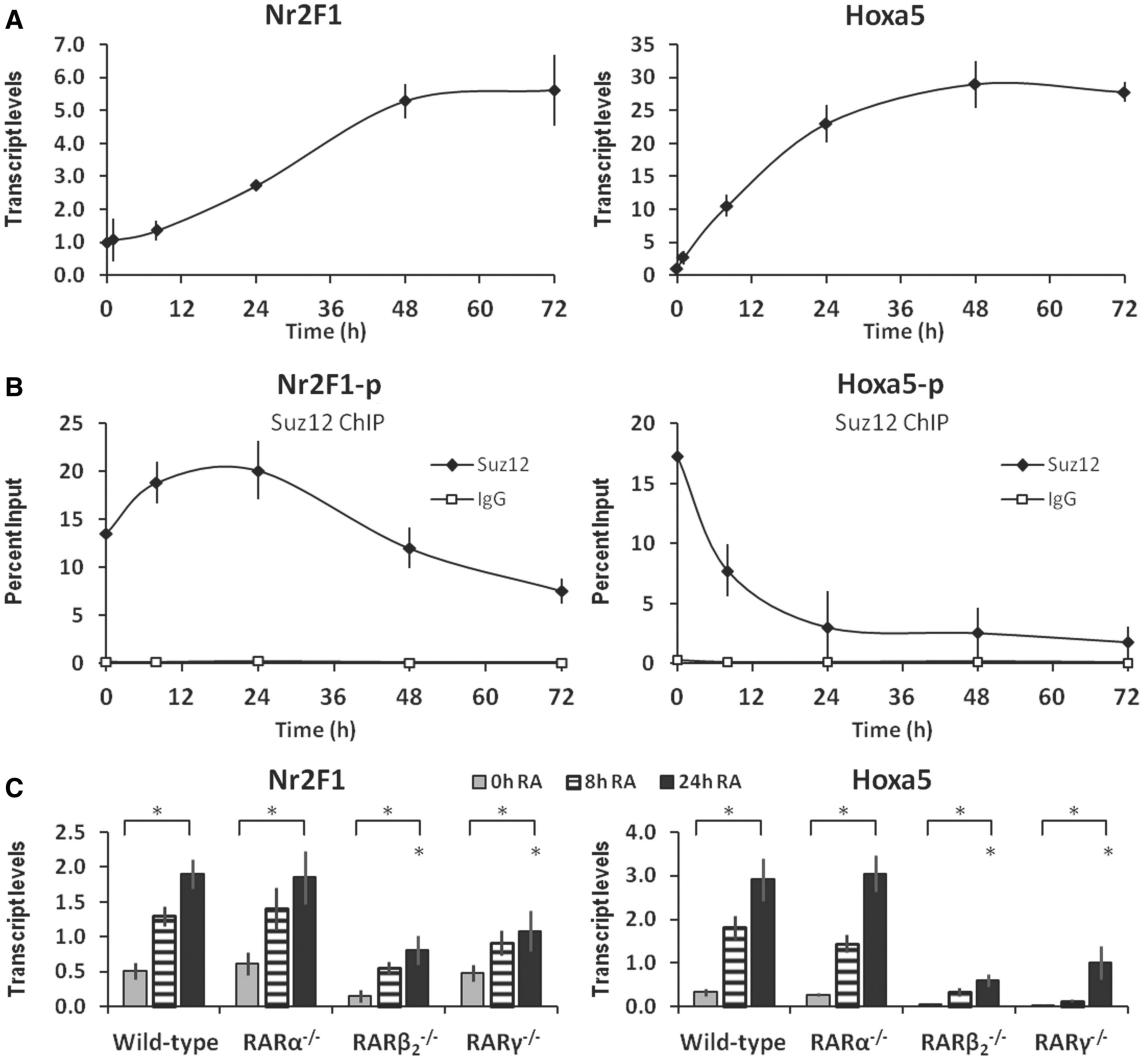
RA increases *Nr2F1* and *Hoxa5* transcript levels in F9 stem cells. (**A**) *Nr2F1* and *Hoxa5* transcript levels in untreated F9 WT cells and on 1, 8, 24, 48 and 72 h of RA treatment (1 μM). (**B**) ChIP analysis shows that the PRC2 core protein component Suz12 initially increases at the *Nr2F1* promoter, but decreases at the *Hoxa5* promoter in response to RA (RefSeq promoter regions). (**C**) *Nr2F1* and *Hoxa5* transcript levels in F9 WT and in RARα, RARβ_2_ and RARγ knockout cells, untreated or treated with RA for 8 or 24 h. Statistical significance (*P* < 0.05) is indicated for the effect of RA (24 h RA relative to vehicle-treated cells), and for the effect of RAR knockout in 24 h RA treatment conditions (relative to WT). All experiments were performed three or more times starting with fresh cells (*n* ≥ 3).

We determined the time-dependent increases in *Nr2F1* and *Hoxa5* transcript levels in response to RA treatment of F9 WT embryonal carcinoma stem cells (Figure 1A). We found that *Nr2F1* transcript levels exhibit a moderate/slow increase, with a half-maximal induction at 24 h, whereas *Hoxa5* transcript levels increase dramatically/rapidly in response to RA, with a half-maximal induction at 12 h. Consequently, the induction of *Nr2F1* is delayed relative to that of *Hoxa5*. We have previously shown that increased transcriptional activity of the *Hoxa-d* clusters in response to RA is associated with decreased PRC2 occupancy of the Hox promoters (23). Therefore, we used ChIP to evaluate the levels of the PRC2 core component protein Suz12 at the *Nr2F1* and *Hoxa5* promoter regions in response to RA treatment of F9 WT cells (Figure 1B). We found that the Suz12 levels at the *Nr2F1* promoter region increased during the first 24 h, and then declined to below the initial levels. In contrast, the Suz12 levels decreased rapidly at the *Hoxa5* promoter region (Figure 1B).

To evaluate the involvement of the RA receptors RARα, RARβ_2_ and RARγ, respectively, we next assessed the *Nr2F1* and *Hoxa5* transcript levels in F9 *RAR* knockout cell lines devoid of individual *RAR* isotypes (21,39–41,53). We found that 24 h of RA treatment significantly increased *Nr2F1* transcript levels in F9 WT (∼4-fold). In the *RARβ_2_^−/−^* and *RARγ^−/−^* cell lines, *Nr2F1* transcript levels were induced by RA to only 43 and 57% of WT levels, respectively (Figure 1C, left). In contrast, the *RARα^−/−^* cell line displayed *Nr2F1* transcript levels similar to those of WT (97%). For comparison, we measured the increase in *Hoxa5* transcript levels in response to RA (Figure 1C, right). The *Hoxa5* gene is directly activated by RA *in vivo* and in cultured ES cells (54). In F9 WT cells *Hoxa5* transcript levels were potently induced by 24 h of RA treatment (∼8-fold). Consistent with *Hoxa5* expression being dependent on RARβ_2_ (54), we found that *Hoxa5* transcript levels were increased to only 20% and 38% of WT levels following RA addition to the *RARβ_2_^−/−^* and *RARγ^−/−^* cell lines, respectively (Figure 1B). In contrast, the *RARα^−/−^* cell line displayed *Hoxa5* transcript levels that were similar to WT (110%). Therefore, we conclude that both RARγ and RARβ_2_ (or downstream targets thereof) are required for the full RA-associated increases in *Nr2F1* and *Hoxa5* transcript levels to be achieved.

### Deletion analysis of the *Nr2F1* upstream region and analysis of DNA methylation status

**Figure 2.**
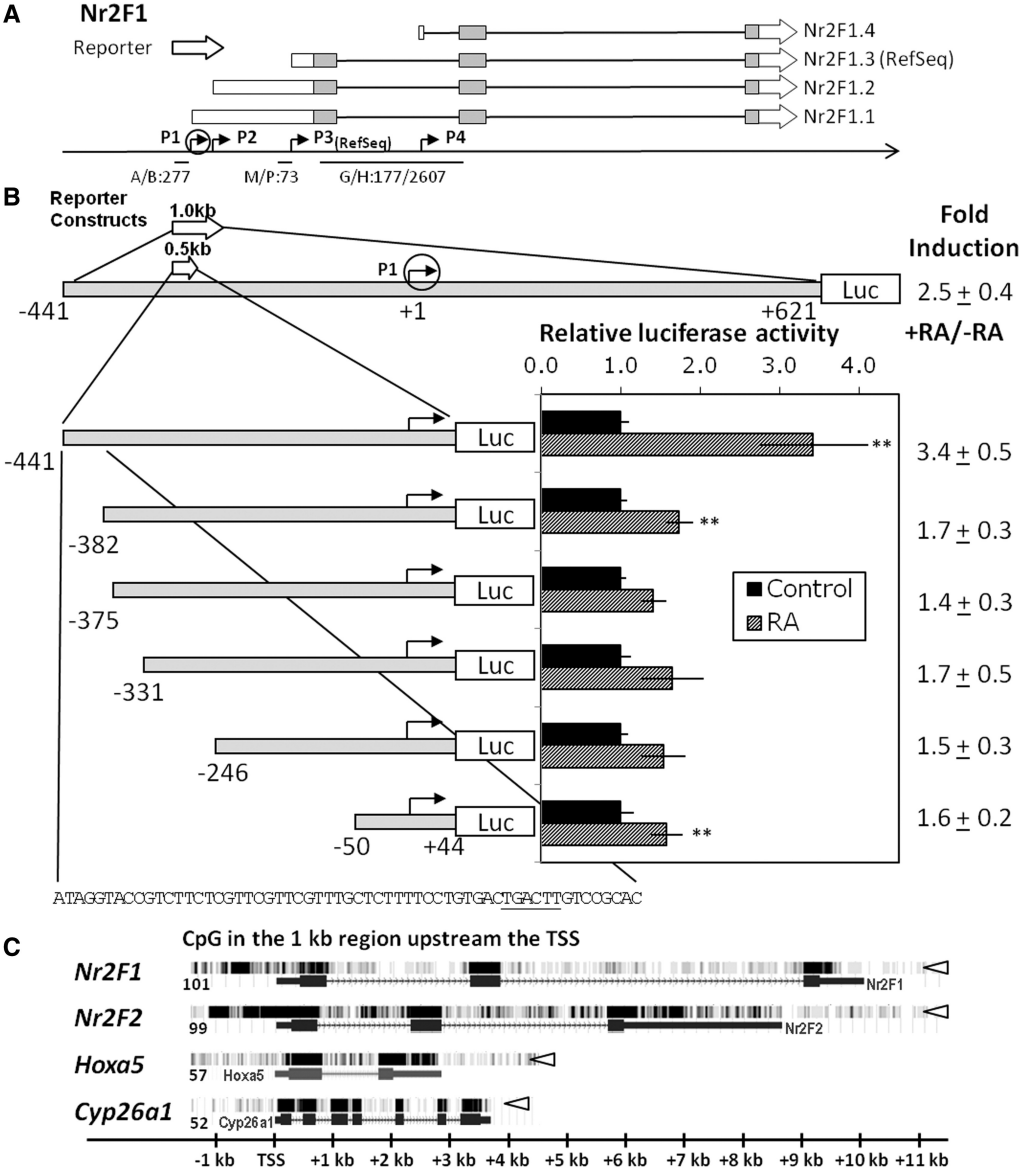
RA responsiveness of the upstream *Nr2F1* promoter region in F9 stem cells. (**A**) Schematic diagram of the murine *Nr2F1* genomic region. RNA transcripts are shown at the top with exons marked by boxes (coding region in gray). Putative promoters are depicted by angled arrows in the genomic map. The most upstream promoter of the mouse *Nr2F1* is specified by a circled arrow. PCR amplicons are marked below (sizes specified in base pairs). Note that for the RT-primers the sizes of both cDNA and gDNA amplicons are indicated. (**B**) RA responsiveness of different regions proximal to the most upstream promoter of the mouse *Nr2F1*. The numbers refer to the 5′ terminal nucleotide included in each construct with respect to the upstream transcription start site (P1-TSS). The nucleotide sequence of the −441 to 382 region, which is required for a potent RA induction (>2-fold), is shown at the bottom of the figure. Note that in the bar diagram only constructs with the P1-TSS located proximal to the luciferase coding region are shown. Luciferase (firefly) reporter constructs containing the indicated regions of the *Nr2F1* sequence were transfected into F9 WT cells, and assayed for RA responsiveness. Relative luciferase activities were expressed as a ratio over the untreated F9 WT cells transfected for each promoter construct, and statistical significance was determined. Data are compiled from at least three independent experiments. **P < 0.05. (**C**) Transcriptional activity at the *Nr2F1*, *Nr2F2*, *Hoxa5* and *Cyp26a1* genes assessed in publically available RNA sequencing data set generated from mouse ES (CCE) cells treated with RA for 5 days (GSM566812) (56). The number of CpG dinucleotides within the 1000 bp sequence upstream of the RefSeq promoter is indicated in the left side of the figure. The *Nr2F1*, *Nr2F2*, *Hoxa5* and *Cyp26a1* reference transcripts are indicated as blue boxes below the RNA-seq intensity data (white arrow heads). Notice the high transcriptional activity extending >1 kb upstream of the *Nr2F1* and *Nr2F2* reference sequence TSSs (P3). This contrasts with the low transcriptional activity upstream of the *Hoxa5* and *Cyp26a1* reference sequence TSSs. The genomic location relative to the RefSeq TSS is indicated at the bottom.

To identify DNA elements required for *Nr2F1* transcriptional activation, we isolated ∼14 kb of the mouse *Nr2F1* locus from a phage library. The phage DNA that contained portions of 5′ *Nr2F1* sequence (55) was digested and fragments were assayed in the pGL3 reporter to identify the DNA region(s) responsible for the RA induction. We identified a 1 kb region upstream of the RefSeq TSS (P3, Figure 2A) as sufficient to drive RA-dependent transcription (data not shown). Importantly, this fragment contains at least two previously identified TSS (P1 and P2, Figure 2A) for the murine *Nr2F1* gene (50,55).

To delineate the region responsible for the RA induction of *Nr2F1*, we generated further deletions of the RA responsive 1 kb construct. Together with a series of shorter deletion constructs, all of which contain the most upstream promoter identified for *Nr2F1* (P1), the 1 kb construct was assayed for RA responsiveness in F9 WT cells (Figure 2B). Using the *Nr2F1* promoter numbering system relative to the P1 promoter described by Salas and colleagues (55), RA increased the reporter activity of the −441 bp promoter/luciferase construct by 3.4-fold (Figure 2A). A 5′-truncation to −382 bp reduced the RA induction to <2-fold. Constructs with further deletions of the *Nr2F1* gene displayed no statistically significant RA induction (Figure 2B). These results suggest the presence in *Nr2F1* of an RA-inducible enhancer element (IEE) positioned at 383–441 bp upstream of the P1 promoter (2.4 kb upstream of the P3 RefSeq promoter).

We next wanted to evaluate the functionality of the P1 promoter in ES cells. Using publically available RNA-seq data, we performed bioinformatic analysis of transcription in RA-treated ES cells (Figure 2C). This analysis revealed transcriptional activity upstream of the *Nr2F1* RefSeq promoter (P3), thereby confirming upstream promoter activity also in ES cells. Interestingly, *Nr2F2* displayed similar transcriptional activity upstream of the RefSeq promoter, whereas both *Hoxa5* and *Cyp26a1* displayed only background levels of transcription upstream of their respective RefSeq promoters (Figure 2C).

DNA methylation, which occurs at CpG dinucleotides, is an epigenetic modification that can potently repress transcription. Regulation by DNA methylation is particularly pronounced in genes with CpG-rich promoter regions. We therefore evaluated the CpG content proximal to the RefSeq TSS of *Nr2F1*, *Nr2F2*, *Hoxa5* and *Cyp26a1*. The 1000 bp regions upstream of the RefSeq TSS of *Nr2F1* and *Nr2F2* displayed a higher density of CpG dinucleotides (∼100 CpGs) as compared with the *Hoxa5* and *Cyp26a1* promoters, which possess a modestly lower CpG density (57 and 52, respectively) as compared with a random 1000 bp sequence (∼62 CpGs) (Figure 2C). As promoter proximal CpG sites are commonly regulated by DNA methylation, we evaluated the DNA methylation status of candidate regions in the *Nr2F1* gene. We found that the *Nr2F1* IEE (P1/P2) and the RefSeq promoter (P3) displayed only minimal DNA methylation both before and after RA treatment of F9 WT cells (Supplementary Figure S2). Thus, changes in DNA methylation are not involved in the changes in *Nr2F1* transcript levels after RA addition.

### RA modifies histone marks and PRC association with the *Nr2F1* and *Hoxa5* genes

**Figure 3.**
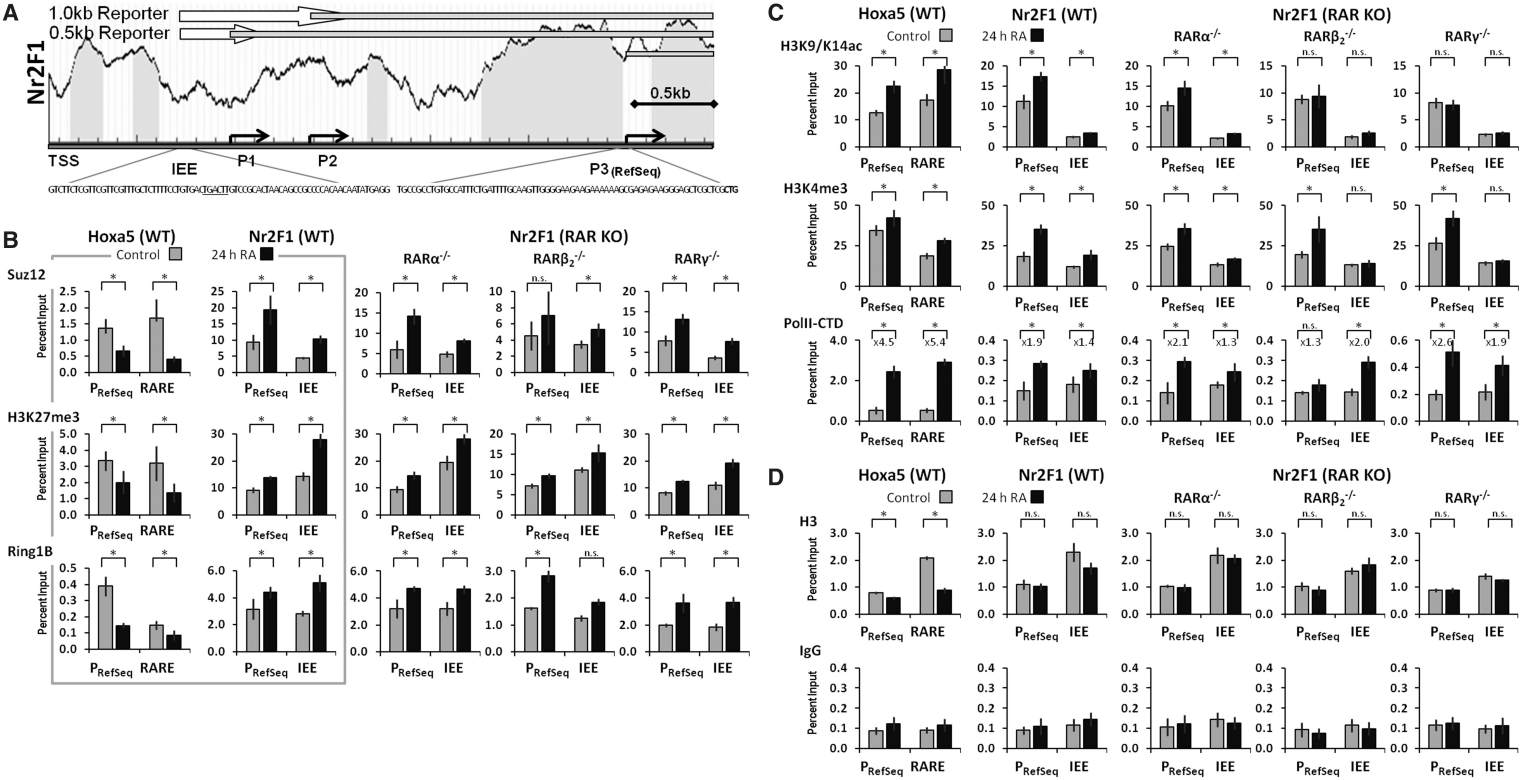
Chromatin Signatures of *Nr2F1* and *Hoxa5* in F9 WT and RAR knockout cells. (**A**) Schematic of *Nr2F1* IEE and promoter regions. *Nr2F1* putative TSSs are indicated by angled arrows (P1, P2 and P3). The proximal nucleotide sequences are shown for the *Nr2F1* promoter and IEE region, and the putative enhancer element is underlined. The GC content of the evaluated regions is illustrated in the background graphs, with CpG islands marked in gray. Note the local depletion of CpGs proximal to the *Nr2F1* RefSeq promoter (P3). White arrows indicate genomic regions included in the *Nr2F1* reporter constructs (Fig. 2B, 0.5 and 1.0 kb, respectively). The gray bars specify primary transcripts from the indicated TSSs. (**B**) ChIP analysis shows that Polycomb repressive marks (Suz12, H3K27me3 and Ring1B) decrease at the *Hoxa5* promoter and RARE, but increase at the *Nr2F1* promoter and IEE in F9 WT cells in response to a 24 h RA treatment (left, boxed). Similar patterns of *Nr2F1* epigenetic changes were observed in RAR knockout cells (KO, right). (**C**) ChIP analysis shows that transcriptional permissive marks (H3K9/K14ac, H3K4me3 and polII-CTD) increase at the *Hoxa5* promoter and RARE, and at the *Nr2F1* promoter and IEE in response to a 24 h RA treatment (left). Similar patterns of *Nr2F1* epigenetic changes were observed in RARα knockout cells, whereas H3K9/14ac levels did not increase in RARβ_2_ and RARγ knockout cells (right). (**D**) ChIP analysis shows Histone 3 occupancy (H3) and the non-specific background signal (IgG). The ChIP signals are depicted relative to the total chromatin input in each ChIP. Statistical significance (*P* < 0.05) is indicated by asterisks for the effect of RA, (n.s.: non-significant).

We next examined the effects of RA on the epigenetic signatures at the *Nr2F1* gene in F9 embryonal carcinoma stem cells. As discussed above, three distinct *Nr2F1* TSSs have been detected in differentiating ES cells (Figures 2C and 3A). We measured the levels of both permissive and repressive histone marks at the IEE (P1/P2) and the RefSeq promoter (P3). To compare *Nr2F1* with a previously identified direct target of RA, we analyzed the epigenetic marks at the *Hoxa5* proximal promoter and retinoic acid response element (RARE) in parallel (54). We found that in F9 WT cells there was an RA-dependent, >2-fold increase in the levels of Suz12 both at the IEE and at the RefSeq promoter (P3) of the *Nr2F1* gene, whereas Suz12 levels were decreased at the *Hoxa5* gene (Figure 3B, left). We observed parallel changes in the PRC2-associated H3K27me3 mark and in the levels of Ring1B, a core component of the PRC1 repressive complex, at both the *Nr2F1* and the *Hoxa5* genes (Figure 3B, left). Notably, for the *Nr2F1* gene, the increase in response to RA was observed at both the RefSeq promoter (P3) and the IEE (P1/P2). Similarly, RA-associated increases in the PRC repressive epigenetic marks occurred in the three F9 *RAR* knockout lines, *RARα^−/−^*, *RARβ_2_^−/−^* and *RARγ^−/−^* at the *Nr2F1* gene (Figure 3B, right). To ascertain that RA was not perturbing the PRC2, we evaluated the association of Suz12 with the PRC2 catalytic subunit EZH2 (Supplementary Figure S3). We found that RA had no effect on the overall interaction between Suz12 and EZH2 core components. Transcriptional activation is often associated with a decrease in epigenetic repressive marks, as we observed for the *Hoxa5* gene (Figure 3B, left), and for *Cyp26a1*, *Hoxa1* and *RARβ_2_* genes (22). Despite the increased levels of PRC repressive marks at the *Nr2F1* RefSeq promoter (P3) and IEE (P1/P2) after RA addition, we observed an increase in *Nr2F1* transcript levels in response to RA (Figure 1A and C).

We next examined the H3K9/K14ac and H3K4me3 histone modifications associated with transcriptional activation and the levels of Ser5-phosphorylated (transcriptionally poised) RNA polymerase II (polII-CTD). The *Nr2F1* RefSeq promoter (P3) displayed increased levels of the H3K9/K14ac and H3K4me3 marks (1.9-fold and 2.1-fold, respectively) on RA treatment of F9 WT cells (Figure 3C, left). Likewise, a 2-fold increase in the polII-CTD level was detected at the *Nr2F1* RefSeq promoter (P3) on a 24 h RA treatment of F9 WT cells (Figure 3C, left). In contrast, we observed only modest changes (<1.5-fold) in the levels of polII-CTD and in the levels of the permissive marks H3K9/K14ac and H3K4me3 at the *Nr2F1* IEE in response to RA treatment (Figure 3C, left). Consequently, the RA-associated increase in transcriptionally permissive marks was more pronounced at the RefSeq promoter (P3) than at the IEE (P1/P2) of the *Nr2F1* gene. At the *Hoxa5* gene, we observed an RA-associated increase in the H3K9/K14ac mark at the promoter and RARE (1.6- and 1.8- fold, respectively), and a large increase in polII-CTD association (>5-fold) with the promoter and the RARE (Figure 3C, left). Also RA-associated increases in the H3K4me3 mark at the promoter and RARE (1.2- and 1.4- fold, respectively) were detected at the *Hoxa5* promoter and RARE (Figure 3C, left). We observed an RA-associated increase in the levels of H3K9/14ac in RARα knockout cells, whereas the H3K9/14ac levels did not increase in RARβ_2_ and RARγ knockout cells (Figure 3C, right). The RA-associated increases in H3K4me3 and polII-CTD at the *Nr2F1* gene in the RAR knockout cells were similar to those observed in F9 WT cells (Figure 3C). The histone density at the *Nr2F1* gene was not affected by RA, whereas RA led to nucleosomal depletion at the *Hoxa5* gene (Figure 3D, bottom). We conclude that the increase in *Nr2F1* transcriptional activity following a 24 h RA treatment of F9 WT cells occurs even when epigenetic repressive factors such as Suz12, Ring1B and the H3K27me3 mark are present in *Nr2F1* regulatory regions.

### Suz12 knockdown reveals that RA differentially regulates the transcription of *Nr2F1* and *Hoxa5* genes

**Figure 4.**
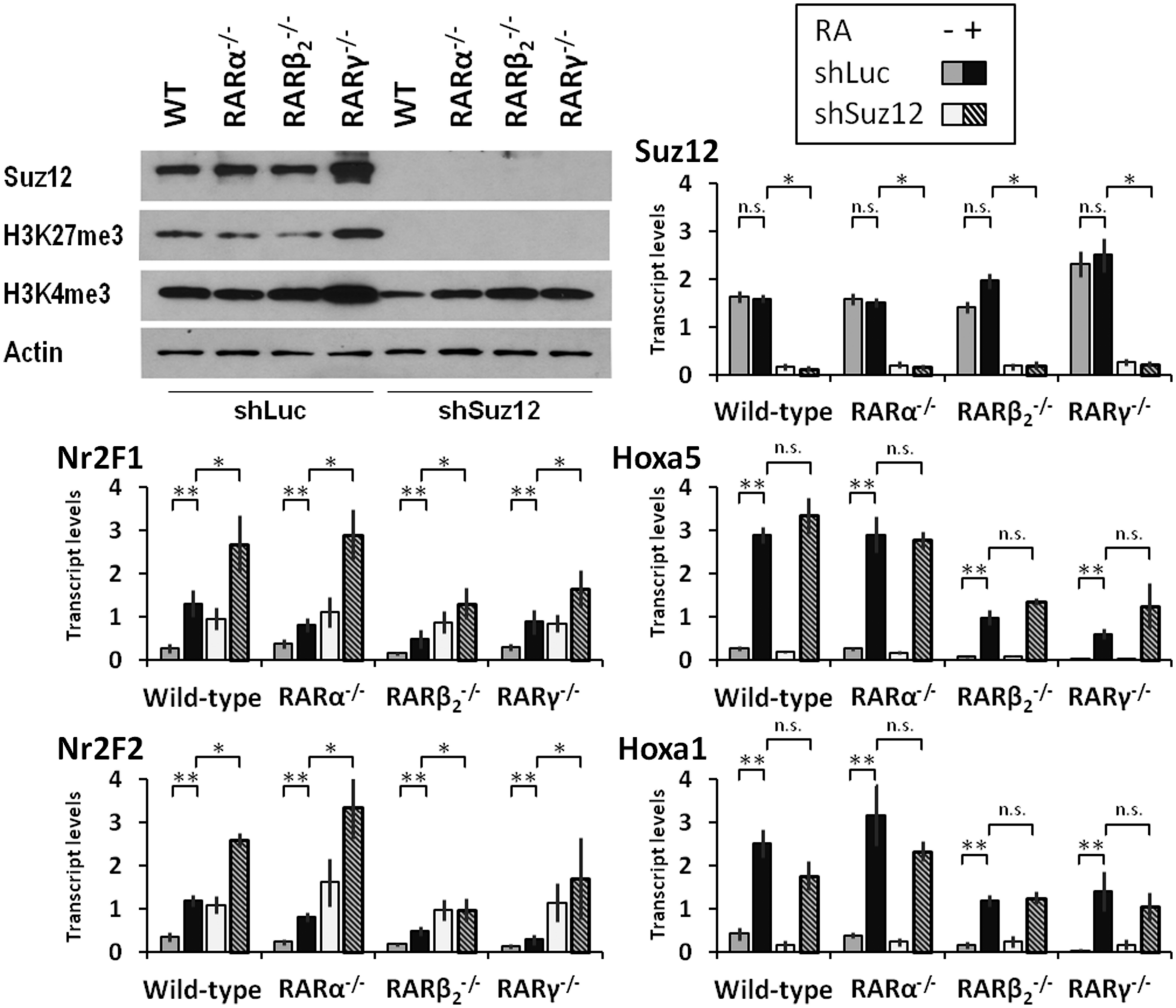
shRNA-mediated Suz12 knockdown increases *Nr2F1* and *Nr2F2* transcript levels. In F9 cells, Suz12 protein and transcript levels (upper left and upper right, respectively) are reduced below detection and to 10%, respectively, by expression of a Suz12-specific shRNA. The Suz12 knockdown eliminates the PRC2-specific histone mark (H3K27me3), but has only a limited effect on the permissive H3K4me3 mark (western blot, upper left). The Suz12 knockdown increases *Nr2F1* (middle left) and *Nr2F2* (bottom left) transcript levels (24 h RA treatment) by quantitative RT-PCR, but has no effect on *Hoxa5* (middle right) and *Hoxa1* (bottom right) transcript levels. Statistical significance is indicated by double asterisk for the effect of RA (relative to vehicle-treated cells), and by asterisk for the effect of Suz12 knockdown in RA-treated conditions (48 h, relative to shLuc control cells).

The RA-induced changes in PRC association with the *Nr2F1* promoter were dramatically different from those of the *Hoxa5* promoter (Figures 1B and 3B), yet both genes display increased transcriptional activity in response to RA (as evident by increased promoter association with permissive histone marks and by increased transcript levels). To define further the role of the Suz12 protein, a key protein component of the PRC2 polycomb repressive complex, we generated knockdown stem cell lines depleted of *Suz12* transcripts. We performed the shRNA-induced Suz12 depletion in the F9 WT cell line and in each of the RAR knockout cell lines. Western blot analyses confirmed that the levels of Suz12 protein (and the levels of K27me3 modified histone 3) in each of the Suz12 knockdown lines were reduced by >90% relative to each of the shLuc (control) transfected parent cell lines (Figure 4). We then evaluated the effects of Suz12 depletion on *Nr2F1*, *Nr2F2*, *Hoxa5* and *Hoxa1* transcript levels in each of these cell lines after 24 h of RA treatment. The Suz12 depletion increased the RA-responsiveness of *Nr2F1* and *Nr2F2* in F9 WT, whereas we detected no significant effects on the RA-responsiveness of *Hoxa5* and *Hoxa1* (Figure 4). In the *RAR* knockout lines, we observed similar effects of Suz12 depletion on these four genes (Figure 4).

**Figure 5.**
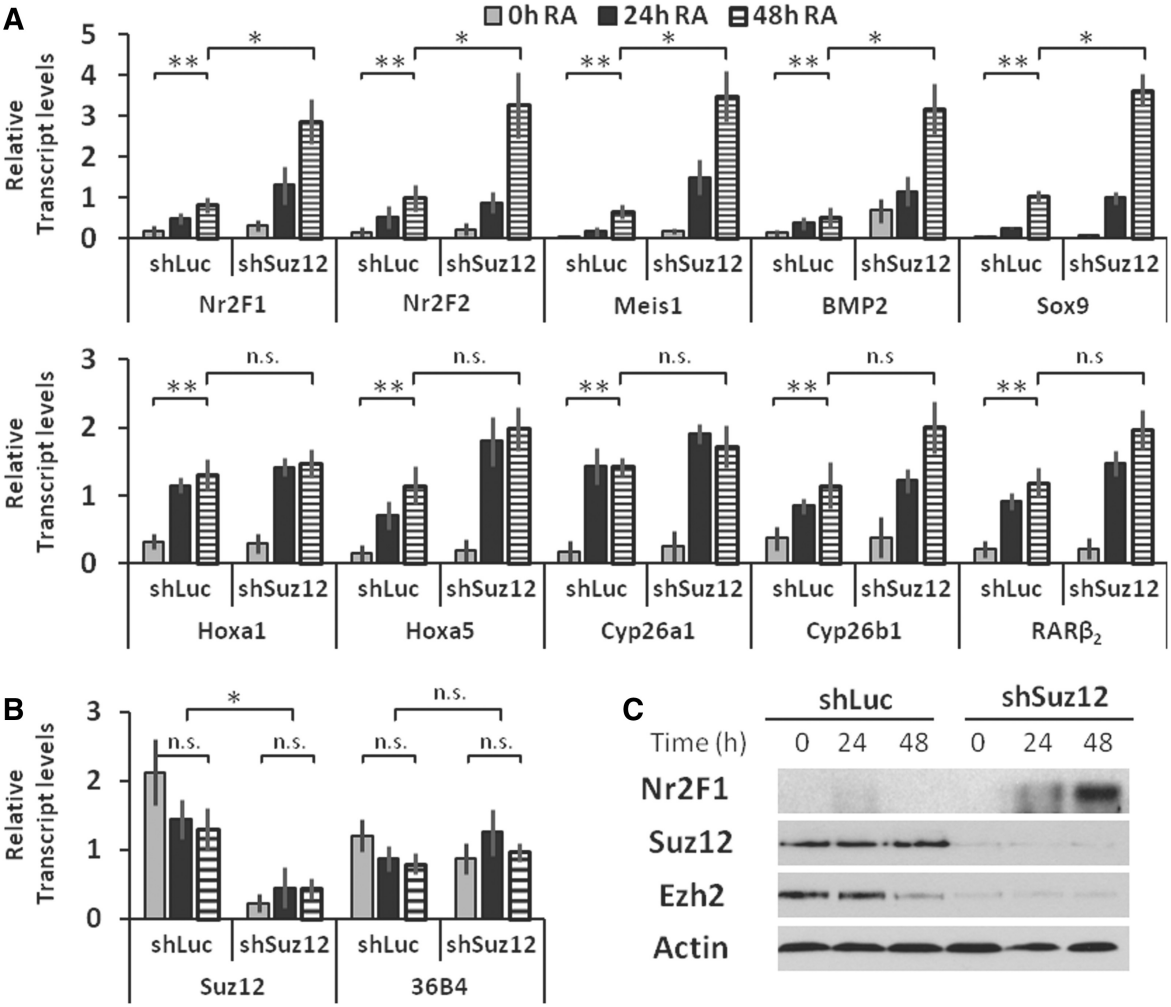
The response to Suz12 depletion distinguishes two classes of RA-inducible genes. (**A**) In ES cells, Suz12 depletion increases *Nr2F1*, *Nr2F2*, *Meis1*, *Sox9* and *BMP2* transcript levels (upper panel), but has no effect on *Hoxa5*, *Hoxa1*, *Cyp26a1*, *Cyp26b1* and *RARβ_2_* transcript levels (lower panel) on RA treatment. Transcripts were measured by quantitative RT-PCR. Statistical significance is indicated by double asterisk for the effect of RA (relative to vehicle-treated cells), and by asterisk for the effect of Suz12 knockdown in RA-treated cells (48 h, relative to shLuc control cells). (**B**) *Suz12* transcript levels are diminished by expression of a Suz12-specific shRNA, whereas transcript levels of the *36B4* reference gene are unaffected. (**C**) The levels of Nr2F1 protein are elevated on Suz12 depletion and increase in an RA-dependent manner by western blotting. The levels of Polycomb proteins Suz12 and Ezh2 are reduced on depletion of Suz12, whereas actin levels remain unchanged by western blotting. Western blots were performed at least three times with different cell extracts; a representative blot is shown.

We next examined the effects of Suz12 depletion on additional RA responsive genes in WT ES cells. We found that the knockdown of *Suz12* by shRNA technology dramatically increased the *Nr2F1*, *Nr2F2*, *Meis1*, *Sox9* and *BMP2* transcript levels in response to RA (Figure 5A). In contrast, *Hoxa1*, *Hoxa5*, *Cyp26a1*, *Cyp26b1* and *RARβ_2_* transcript levels were not significantly affected by the Suz12 depletion in both control and RA-treated cells (Figure 5A). The levels of *Suz12* transcripts in ES cells stably transfected with shSuz12 constructs were significantly reduced compared with the levels in shLuc (control) transfected ES cells (Figure 5B). As expected, the transcript levels of the *36B4* reference gene did not change in response to RA (Figure 5B). An RA-dependent increase in Nr2F1 protein was observed on Suz12 depletion, whereas the levels in control cells were below detection (Figure 5C). The western blot analysis also confirmed that the total levels of Suz12 protein do not change in response RA. Furthermore, the Suz12 knockdown reduced protein levels by >90% relative to control ES cells (Figure 5C). The depletion of Suz12 also reduced the levels of EZH2 protein (Figure 5C).

Overall, our data suggest differential PRC2 dynamics in RA-induced transcription of the *Nr2F1*, *Nr2F2*, *Meis1*, *Sox9* and *BMP2* genes versus *Hoxa5*, *Hoxa1*, *Cyp26a1*, *Cyp26b1* and *RARβ_2_* genes. Specifically, Suz12 exhibits an inhibitory effect on the RA-associated increases in *Nr2F1*, *Nr2F2*, *Meis1*, *Sox9* and *BMP2* transcript levels, but not on the RA-associated increase in *Hoxa5*, *Hoxa1*, *Cyp26a1*, *Cyp26b1* and *RARβ_2_* transcript levels (Figure 5A). Importantly, whereas the *Nr2F1* promoter shows increased PRC2 association on RA treatment (Figures 1B and 3B), the *Hoxa5*, *Hoxa1*, *Cyp26a1* and *RARβ_2_* promoters all respond to RA addition by dissociation of the PRC2 component (Figures 1B and 3B and previous publications), thus potentially explaining the more rapid transcriptional induction by RA of this latter group of genes.

### *In Silico* ChIP-seq analysis of RARγ, RXRα and RNA polII association in F9 WT cells

A recently published ChIP-seq analysis of RARγ, RXRα and RNA polII in F9 WT cells (57) allowed us to expand our analysis of the epigenetic signature of RA responsive genes. We analyzed the RARγ, RXRα and RNA polII chromatin association with the PRC2-attenuated RA target genes *Nr2F1, Nr2F2* and *Sox9*, and with the non-attenuated RA target genes *Hoxa5* and *Cyp26a1* at various time points (Supplementary Figure S4). The ChIP-seq analysis confirmed recruitment of RNA polII to the *Nr2F1*, *Nr2F2*, *Sox9*, *Hoxa5* and *Cyp26a1* proximal promoter regions in response to RA treatment (Supplementary Figure S4). Importantly, with respect to *Hoxa5* and *Cyp26a1*, RNA polII was not only recruited to the proximal promoter regions in response to RA but was also detected distributed throughout the length of each gene, consistent with highly active transcription. In contrast, recruitment of RNA polII appeared to be lower at both the promoters (as observed in Figure 3C) and throughout the *Nr2F1*, *Nr2F2* and *Sox9* genes in the presence of RA (Supplementary Figure S4).

The co-localization of RARγ and RXRα at specific genomic locations is strong evidence for an RARE. Indeed, the ChIP-seq analysis confirmed the previously identified RAREs in *Hoxa5* and *Cyp26a1* (indicated in Supplementary Figure S4). The *Cyp26a1* distal RARE showed association with both RARγ and RXRα, whereas the proximal RARE was predominantly associated with RXRα. This may indicate that the proximal RARE is primarily bound by RARα or RARβ, whereas the distal RARE is primarily bound by RARγ. Neither RARγ nor RXRα was associated with the *Nr2F1*, *Nr2F2* and *Sox9* genes (Supplementary Figure S4). This suggests that *Nr2F1*, *Nr2F2* and *Sox9* are secondary targets in the RA signaling cascade. Alternatively, *Nr2F1*, *Nr2F2* and *Sox9* could be targeted by different RAR and RXR isotypes (e.g. RARα or RARβ dimerized with RXRβ or RXRγ).

## DISCUSSION

**Figure 6.**
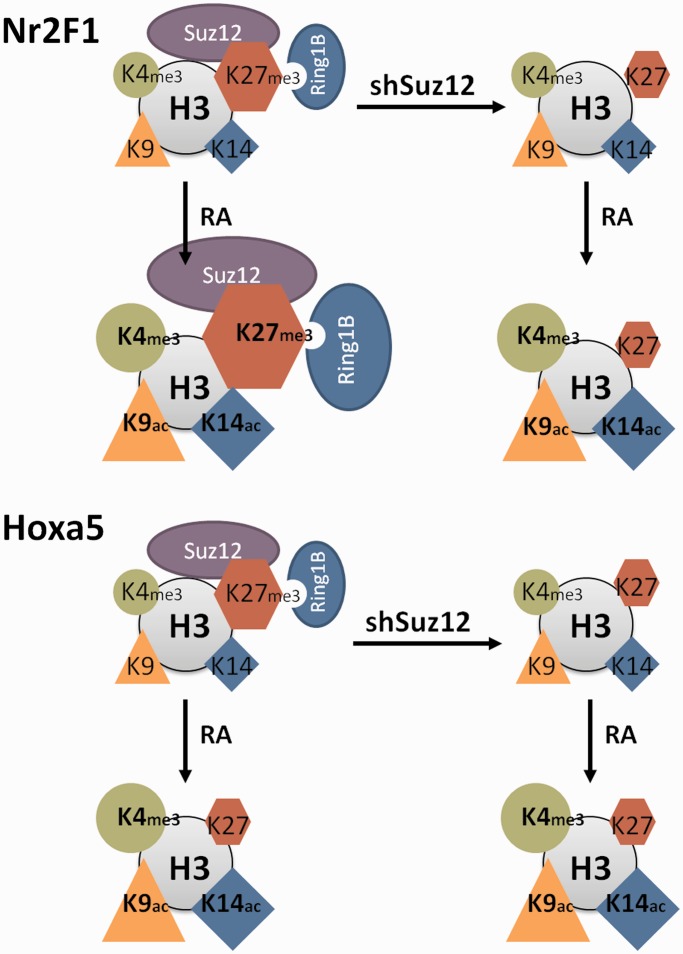
Summary model of *Nr2F1* and *Hoxa5* epigenetic signatures in response to RA. *Nr2F1* and *Hoxa5* display different epigenetic signatures on RA treatment of stem cells. *Nr2F1* is characterized by increased levels of PRC (Suz12 and Ring1B) and the associated H3K27me3 histone mark in response to RA. In contrast, the *Hoxa5* epigenetic signature is characterized by dissociation of PRC (Suz12 and Ring1B) and reduction of the H3K27me3 histone mark. Note that the epigenetic signatures of *Hoxa5* are similar in presence and absence of Suz12 after RA addition. In contrast, the epigenetic signature of *Nr2F1* on RA treatment differs in the presence and absence of Suz12, thus potentially explaining the increased transcriptional activity on Suz12 knockdown. For both *Nr2F1* and *Hoxa5*, the transcriptional induction is marked by increased levels of H3K4me3, H3K9ac and H3K14ac permissive histone marks. The different marks are depicted as shapes whose sizes reflect the relative abundance in the specified condition. Histone H3 is depicted as a gray circle. PRC1 and PRC2 are represented by Ring1B and Suz12, respectively.

In the current study, we have identified a novel class of PRC target genes, represented by *Nr2F1*, *Nr2F2*, *Meis1*, *Sox9* and *BMP2*, that is activated during RA-induced stem cell differentiation. The RA-associated transcriptional activation of *Nr2F1* occurs along with both increases in PRC and H3K27me3 repressive marks and permissive histone marks such as H3K4me3. The differential effects of Suz12 depletion on *Nr2F1*/*Nr2F2* and *Hoxa5*/*Hoxa1* (Figures 5 and 6) point to PRC2 dynamics as a distinguishing feature between the canonical direct RA target genes (rapidly induced) and a class of attenuated RA target genes (slowly induced). PRC2 was identified as a repressor of Hox gene transcription in *Drosophila* (58,59). It it was thus somewhat surprising that functional depletion of PRC2 in murine ES cells did not enhance transcription activation of *Hoxa1* (61) or *Hoxa5* (Figure 4). The functional characterization of PRCs is further complicated by the variable PRC complex composition and the numerous PRC target genes (62,63). Several groups have evaluated individual polycomb components (10,64), and Pasini et al. established a role for the Suz12 polycomb protein in ES cell differentiation (8). They further identified distinct groups of PRC-regulated genes: (i) genes expressed in pluripotent ES cells and silenced in differentiated cells, (ii) genes transcriptionally activated on differentiation and (iii) genes that were initially activated and then silenced in differentiated cells. This classification was corroborated by a recent report in which Mendoza-Parra et al. distinguished between (i) genes silenced by RA, (iia) genes rapidly induced by RA and (iib) genes exhibiting delayed induction by RA (57). This genome-wide ChIP-seq study involved algorithm-based grouping of RA responsive genes, thereby providing a blinded validation of the grouping. The emerging consensus is that genes expressed after differentiation show strong polycomb protein association in the pluripotent stem cell state, whereas genes expressed in stem cells show strong polycomb association in the differentiated state (8,57). Our findings, however, reveal that this broad consensus does not fit genes that display a delayed induction in response to RA (e.g. *Nr2F1* and *Nr2F2*). We show here that such genes initially display an increase in PRC2 levels concurrent with transcriptional activation. We demonstrate that PRC2 attenuates transcription of *Nr2F1* and describe the associated epigenetic changes (Figures 4–6). This provides new mechanistic insights into genes exhibiting delayed induction by RA i.e. *Nr2F1* (iib) and genes rapidly induced by RA i.e. *Hoxa5* (iia) during stem cell differentiation.

To understand better the regulatory regions and chromatin environment of the *Nr2F1* gene, we compared the local epigenetic contexts of the *Nr2F1* and *Nr2F2* genes with those of *Cyp26a1* and *Hoxa5*. The *Nr2F1*, *Nr2F2*, *Cyp26a1* and *Hoxa5* genes are embedded within bivalent chromatin regions associated with CpG islands (Figure 3 and Supplementary Figure S5), as is typically seen for genes, with the potential for transcriptional activation or repression depending on the ES cell differentiation state (4,65). The *Nr2F1* and *Nr2F2* promoter regions are enriched for CpG sites relative to the *Cyp26a1* and *Hoxa5* promoter regions (Figure 2C). Mammalian promoters show a bimodal distribution based on CpG content (66,67), and blinded computational analyses have confirmed that key epigenetic histone marks differ among promoters of high versus low CpG content (68). RA resulted in enrichment of PRC proteins at the *Nr2F1* promoter (high CpG content), but resulted in a decrease in PRC proteins located at the *Hoxa5* promoter (low CpG content). This difference could be related to the presence or absence of a TATA-box, which specifies a distinct TSS only in promoters with low CpG content (69). The observation that *Nr2F1* and *Nr2F2* use loosely defined TSSs, whereas *Cyp26a1* and *Hoxa5* each uses a clearly defined TSS (Figure 2C), further supports this grouping of genes into different functional classes.

Differences in the epigenetic environment of the *Nr2F1*/*Nr2F2* versus the *Cyp26a1*/*HoxA5* genes in untreated conditions could potentially explain the differential response to RA, i.e. slow versus rapid induction (Figure 1A). In the absence of RA (untreated ES cells), *Nr2F1*, *Nr2F2*, *Cyp26a1* and *HoxA5* are transcriptionally silent (Figure 5). We therefore compared the epigenetic signatures in untreated ES cells using publicly available ChIP-seq data (Supplementary Figure S4). The key role of PRC2 (Figures 1B and 3B) prompted us to further evaluate PRC2 associated epigenetic marks (H3K27me3, EZH2, Suz12 and Ring1B) and H3K4 methylation of *Nr2F1*, *Nr2F2*, *Cyp26a1* and *HoxA5* genes. Our bioinformatics analysis suggests that in the absence of RA, the epigenetic distribution of H3K27me3 and PRC1/2 is lower at the *Nr2F1* and *Nr2F2* genes in comparison with the Cyp26a1 and HoxA5 genes in ES cells. The RefSeq promoter regions of *Nr2F1* and *Nr2F2* show a localized decrease of H3K27me3 on either side of the TSS, which extends to a localized decrease in H3K4 methylation. In contrast, in the absence of RA, the *Hoxa5* and *Cyp26a1* proximal promoter regions show high levels of H3K27me3 and H3K4me3 and decreased H3K4 methylation proximal to the TSS, which may be the result of nuclesome depletion around the TSS. Interestingly, RNA-seq data (Figure 2C) suggest that whereas the TSSs of *Hoxa5* and *Cyp26a1* are clearly defined, *Nr2F1* and *Nr2F2* each may use a number of alternative sites for transcriptional initiation. Consistent with these data, the RA responsive region of *Nr2F1* (IEE, Figure 2) is enriched for the H3K4me1 modification (70), progressively transitioning on RA addition to H3K4me2/3, which co-localizes with H3K27me3 and Jarid2a upstream of the TSS (Supplementary Figure S4). These features are similar to the features recently described for PRC-associated permissive enhancers (14), which permit cell type–specific transcriptional activation of PRC-repressed stem cell genes depending on the promoter context. The attenuated induction of *Nr2F1* may involve recruitment of Ring1B and/or Jarid2, which has been reported to introduce pausing of polII at loci primed for future transcriptional activation (64,71). Indeed, we found that Ring1B was recruited to the IEE and RefSeq promoter of *Nr2F1* in response to RA (Figure 3B). The *RARβ_2_* and *RARγ* knockout cell lines displayed reduced induction of *Nr2F1* by RA (Figure 1C). However, polII was recruited even more efficiently in *RARγ^−/−^* cells than in WT cells (Figure 3C, right). One explanation could be that the absence of RARβ_2_ reduces polII recruitment, whereas the absence of RARγ affects the initiation of transcriptional elongation. The RA-associated increase in H3K4me3 levels observed at the *Nr2F1* gene in both the RARβ_2_ and RARγ knockout cell lines suggests that RA induces a partially permissive chromatin structure (Figure 3C). In contrast, we observed no increase in H3K9/14ac levels at the *Nr2F1* gene in the RARβ_2_ and RARγ knockout cell lines, which suggests that histone acetylation is required for full transcriptional activation of *Nr2F1*.

The role of Nr2F1 in RA-associated endodermal differentiation of ES cells (35) and the potential of RA to function as a ligand for Nr2F2 (37), and as a regulator of mesenchymal differentiation (38) point to the Coup-TFs as key players in RA-induced differentiation. *Nr2F1* basal transcription is mediated by three ETS response elements (55), yet the DNA elements within the *Nr2F1* promoter that mediate the RA responsiveness of *Nr2F1* are unknown (27,34). The *Nr2F1* promoter region contains no consensus RARE (DR2 or DR5), and thus far no functional element has been identified that can explain the RA responsiveness of *Nr2F1* (Supplementary Figure S4). This is the first study identifying the *Nr2F1* promoter region that is responsible for the RA induction. The complex dynamics of PRC association in response to RA suggest that several transcription factors cooperate to regulate the transcription of the *Nr2F1* gene. Thus, it will be important to identify the *cis*-regulatory DNA elements responsible for the RA-associated PRC recruitment and to characterize the transcription factors that recognize these DNA elements.

Here we identify PRC2 dynamics as a distinguishing feature between two classes of PRC2 target genes represented by the *Nr2F1* (*Coup-TF1*) and the *Hoxa5* gene, respectively. We conclude that PRC1/2, in addition to specifying transcriptional repression, can function to attenuate transcriptional activation by RA of specific genes during stem cell differentiation. Attenuation of the maximal transcriptional activation may allow for more exquisite precise regulation of commitment to a specific differentiation pathway.

## SUPPLEMENTARY DATA

Supplementary Data are available at NAR Online: Supplementary Tables 1 and 2 and Supplementary Figures 1–5.

## FUNDING

National Institutes of Health (NIH) [R01 CA043796 to L.J.G.]; WCMC funds; NIH [T32 CA062948 to Y.Z. for a portion of this research]; Fonds de Recherche en Santé du Québec [PF1-Benoit-25389 to Y.D.B]. Funding for open access charge: The University of Nottingham's open access publishing fund (pending approval).

*Conflict of interest statement.* None declared.

## Supplementary Material

Supplementary Data
