# Supplementary material for: Polycomb recruitment attenuates retinoic acid–induced transcription of the bivalent NR2F1 gene
Source: Nucleic Acids Res. 2013 May 10;41(13):6430–43. doi: 10.1093/nar/gkt367 (PMC3905905; doi:10.1093/nar/gkt367)
Supplement: Supplementary Data [file supp_41_13_6430__index.html]

Polycomb recruitment attenuates retinoic acid–induced transcription of the bivalent NR2F1 gene — Supplementary Data 

# Polycomb recruitment attenuates retinoic acid–induced transcription of the bivalent *NR2F1* gene

## Supplementary Data

files

**Files in this Data Supplement:**

- Supplementary Data - pdf file
- Supplementary Data - Laursen\_Supp\_FINAL.pdf
