# Supplementary material for: Polycomb recruitment attenuates retinoic acid–induced transcription of the bivalent NR2F1 gene
Source: Nucleic Acids Res. 2013 May 10;41(13):6430–43. doi: 10.1093/nar/gkt367 (PMC3905905; doi:10.1093/nar/gkt367)
Supplement: Supplementary Data [file supp_gkt367_nar-00794-x-2013-File008.pdf]

SUPPLEMENTARY INFORMATION

ABBREVIATIONS

cDNA:	complementary DNA
Coup-TF:	Chicken Ovalbumin Upstream Promoter-Transcription-Factor
ES:	Embryonic Stem
gDNA:	genomic DNA
H3K27me3:	Trimethylated Histone 3 Lysine 27
H3K4me3:	Trimethylated Histone 3 Lysine 4
IEE:	Inducible Enhancer Element
NR2F1:	Nuclear Receptor 2F1
polII:	RNA polymerase 2
PRC:	Polycomb Repressive Complex
RA:	all-trans Retinoic Acid
RAR:	Retinoic Acid Receptor
RARE:	Retinoic Acid Responsive Element
RefSeq:	NCBI Reference Sequence
RT:	Reverse Transcription
RXR:	Retinoid X Receptor
TSS:	Transcriptional Start Site
WT:	Wild-Type

FIGURE LEGENDS

Figure S1. Genotypic validation of F9 RAR knockout cell lines.
Transcript levels were assessed in triplicate samples after 0, 8 and 24 hours treatment with RA. The RAR isoform and PCR band sizes are indicated to the left of the gel. Note that RARβ$_2$ is itself induced by RA. The truncated transcripts detected in RARα and RARγ knockout cell lines, respectively, represent nonsense coding mRNAs resulting from the genomic manipulations. The three F9 RAR knockout cell lines were all generated by former lab members (39-41).

Figure S2. CpG methylation of *Nr2F1* RARE and promoter regions. The CpG methylation is low at the enhancer and at the promoter regions of *Nr2F1* both in the absence (blue) and in the presence (red) of RA. Each horizontal line represents the methylation status of an independent allele. The numbers below the figures indicate the CpG position relative to the P_{RefSeq} transcriptional start site (+1).

Figure S3. Co-IP of PRC2 core component Ezh2. Suz12 interacting protein Ezh2 was immunoprecipitated using a Suz12 directed antibody and detected by Western blot analysis. Ezh2 associated with Suz12.

Figure S4. The Effect of RA on RARγ, RXRα, and PolII association with *Nr2F1, Nr2F2, Sox9, Hoxa5,* and *Cyp26a1.* RARγ, RXRα, and PolII association were characterized using publically available genome wide chromatin immunoprecipitation assays coupled with next generation DNA sequencing datasets (ChIP-seq). **(A)** *Nr2F1*, **(B)** *Nr2F2,* **(C)** *Sox9*, **(D)** *Hoxa5*, and **(E)** *Cyp26a1* genomic loci in mouse F9 stem cells. ChIP-seq for untreated (0 h RA) and RA treated F9 cells (6 h and 48 h RA) are shown for RARγ (purple), RXRα (green), and RNA polII (blue). Each read is aligned to the corresponding genomic location, thereby visualizing specific

chromatin association as density of reads. RefSeq intron and exon locations are specified in blue at the bottom of each alignment. Chromosome coordinates and scale bars are located at the top of each alignment.

Figure S5. The Epigenetic signatures of *Nr2F1, NR2F2, Hoxa5,* and *Cyp26a1*. Histone modifications and PRC component association were characterized using publically available genome wide chromatin immunoprecipitation assays coupled with next generation DNA sequencing datasets (ChIP-seq). **(A)** *Nr2F1*, **(B)** *Nr2F2,* **(C)** *Hoxa5* and **(D)** *Cyp26a1* genes in mouse embryonic stem cells. Accession numbers for datasets used were for ChIPseq: F9 RARγ, F9 RXRα, F9 Pol II (GSE30538); ES H3K4me3, ES H3K27me3, ES RNA pol II (GSE12241); ES H3K4me1, ES H3K4me2 (GSE11172); PRC proteins, ES Suz12, ES EZH2, ES Ring1B (GSE13084); ES H3K27ac (GSE24165); Jarid2 (GSE465889). RNAseq: mRNA expression analysis of RA differentiated mouse ES cells (GSM566812). DNA methylation and hydroxymethylation state (GSE28682).

FIGURES

Figure S1

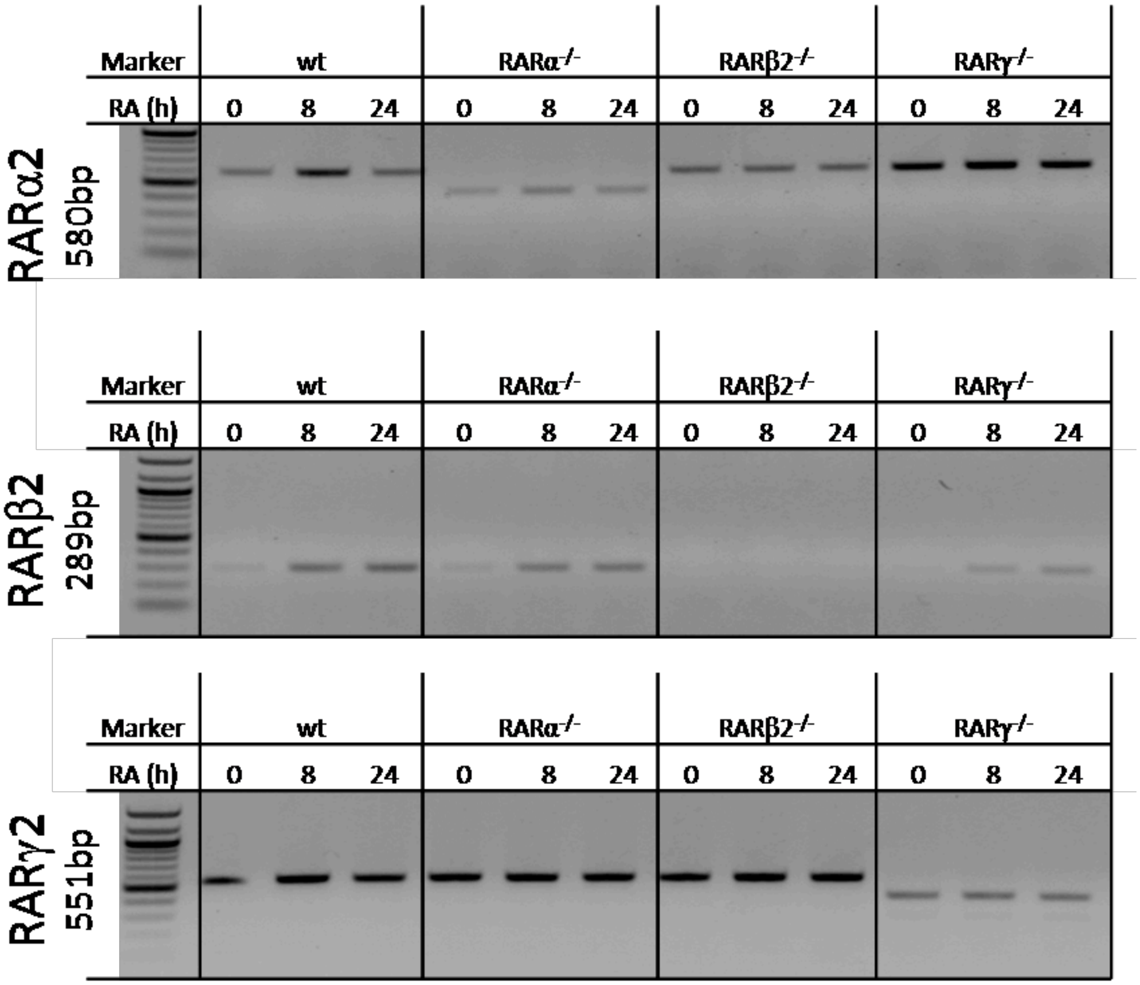

Figure S2

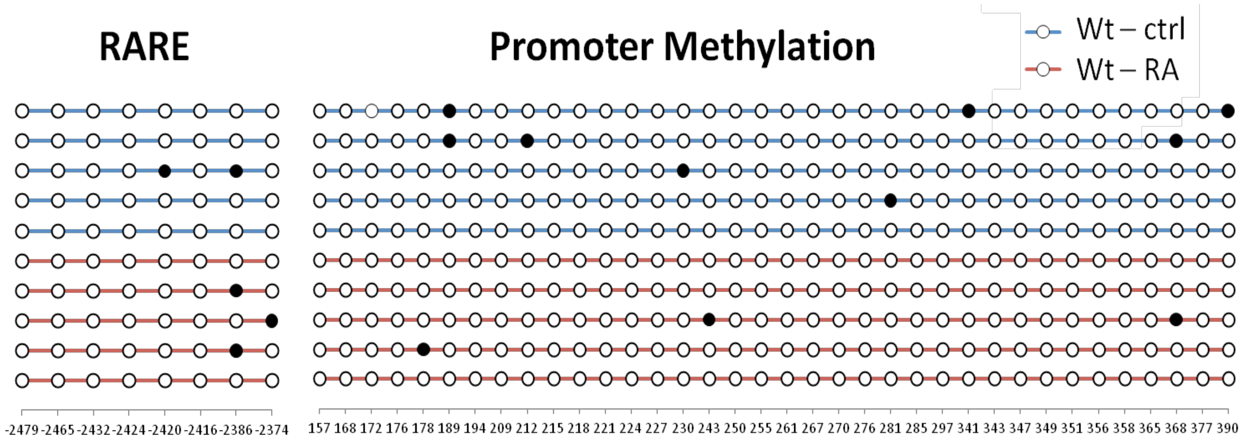
RARE
Promoter Methylation
Wt – ctrl
Wt – RA
-2479 -2465 -2432 -2424 -2420 -2416 -2386 -2374
157 168 172 176 178 189 194 209 212 215 218 221 224 227 230 243 250 255 261 267 270 276 281 285 297 341 343 347 349 351 356 358 365 368 377 390

Figure S3

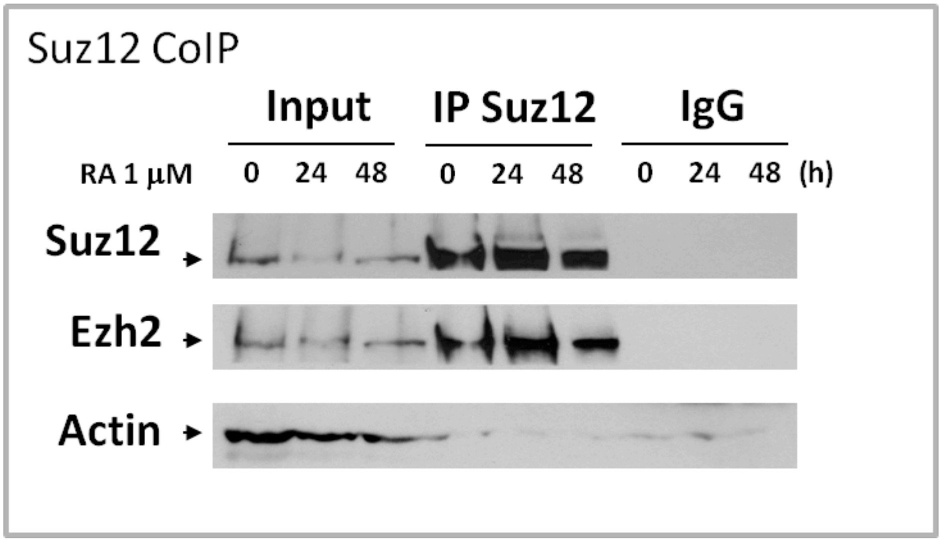
Suz12 CoIP
Input
IP Suz12
IgG
RA 1 μM
0 24 48 0 24 48 0 24 48 (h)
Suz12
Ezh2
Actin

Figure S4

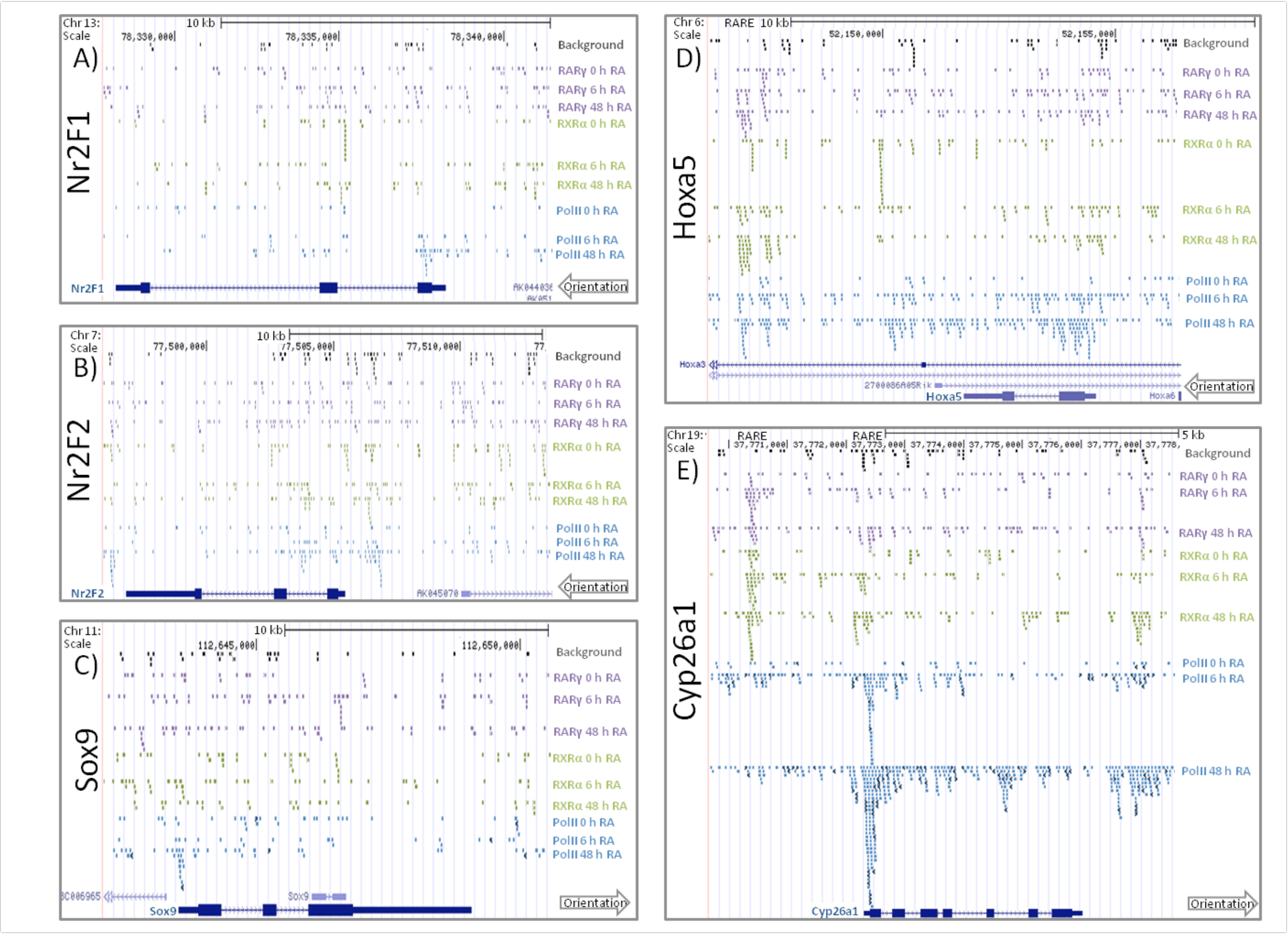

A)
Nr2F1
Chr 13:
Scale
10 kb
78,330,000
78,335,000
78,340,000
Background
RARγ 0 h RA
RARγ 6 h RA
RARγ 48 h RA
RXRα 0 h RA
RXRα 6 h RA
RXRα 48 h RA
PolII 0 h RA
PolII 6 h RA
PolII 48 h RA
Nr2F1
Orientation
B)
Nr2F2
Chr 7:
Scale
10 kb
77,500,000
77,505,000
77,510,000
Nr2F2
AK045070
C)
Sox9
Chr 11:
Scale
10 kb
112,645,000
112,650,000
Sox9
D)
Hoxa5
Chr 6:
RARE
10 kb
52,150,000
52,155,000
Hoxa3
Hoxa5
Hoxa6
E)
Cyp26a1
Chr 19:
RARE
5 kb
Cyp26a1

Figure S5

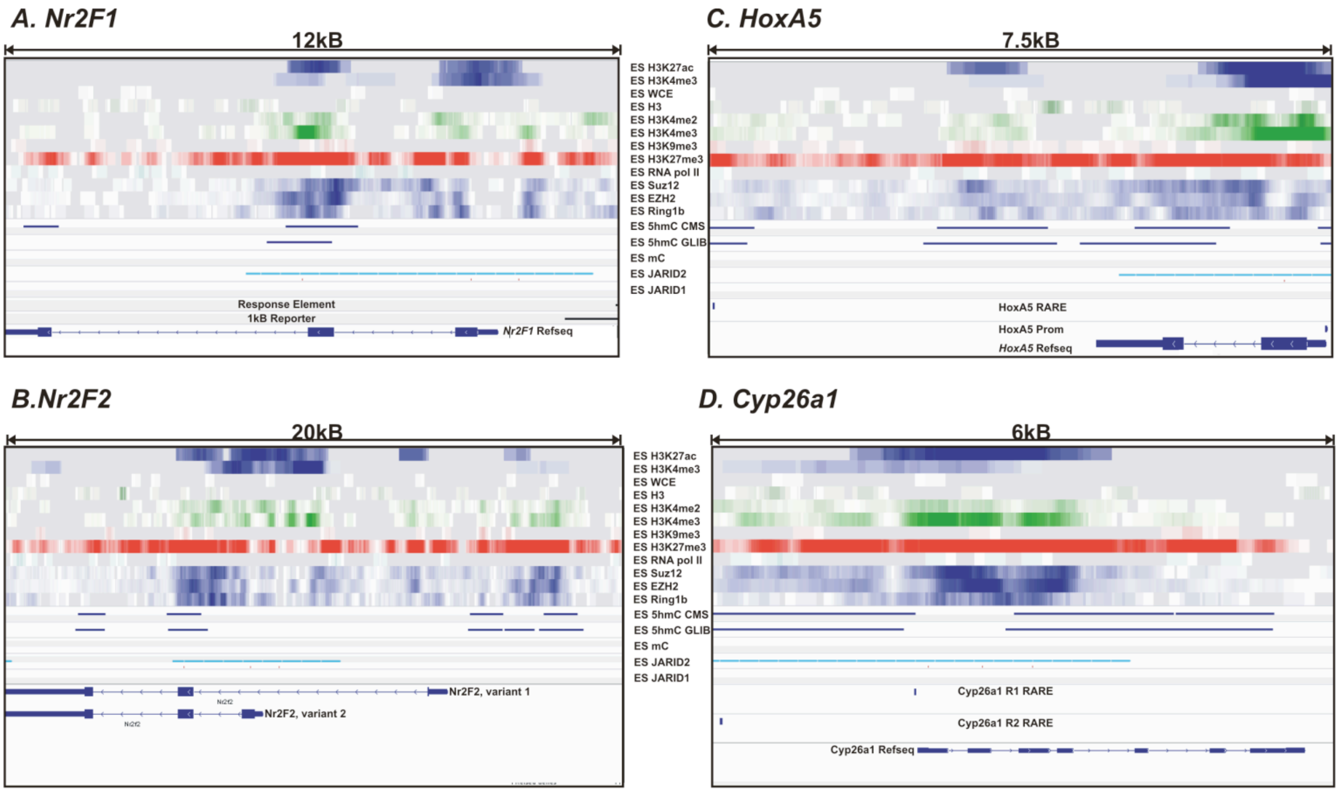

A. Nr2F1
12kB
C. HoxA5
7.5kB
B.Nr2F2
20kB
D. Cyp26a1
6kB
ES H3K27ac
ES H3K4me3
ES WCE
ES H3
ES H3K4me2
ES H3K4me3
ES H3K9me3
ES H3K27me3
ES RNA pol II
ES Suz12
ES EZH2
ES Ring1b
ES 5hmC CMS
ES 5hmC GLIB
ES mC
ES JARID2
ES JARID1
Response Element
1kB Reporter
Nr2F1 Refseq
HoxA5 RARE
HoxA5 Prom
HoxA5 Refseq
Nr2F2, variant 1
Nr2F2, variant 2
Cyp26a1 R1 RARE
Cyp26a1 R2 RARE
Cyp26a1 Refseq

Table S1: Gene Expression Primers

**Forward**	Sense primer (5'-3')	**Reverse**	Antisense primer (5'-3')	**Product**	(bp)
Induced by RA				cDNA	gDNA
mBMP2(+)A	CTTAGACGGACTGCGGTCTCCTA	mBMP2(-)B	ACGTCTTCCGAAGGCCGGGACA	171	1238
mCyp26a1(+)A	GAAACATTGCAGATGGTGCTTCAG	mCyp26a1(-)B	CGGCTGAAGGCCTGCATAATCAC	272	728
mCyp26b1(+)A	TGGACTGTGTCATCAAGGAGGT	mCyp26b1(-)B	GTCGTGAGTGTCTCGGATGCTA	143	492
mHoxa1(+)E	TAACTCCTTATCCCCTCTCCAC	mHoxa1(-)D	ACCCACGTAGCCGTACTCTCCA	151	628
mHoxa5(+)C	CCCCTGGATGCGCAAGCTGCACATT	mHoxa5(-)F	TTCTCCAGCTCCAGGGTCTGGTAGCGA	105	1062
mMeis1(+)A	CATGATAGACCAGTCCAACC	mMeis1(-)D	GGCTACATACTCCCCTGGCATACT	243	3827
mNR2F1(+)K	AGCCATCGTGCTATTCACG	mNR2F1(-)L	TTCTCACCAGACACGAGGTC	570	5718
mNR2F1(+)G	CTGTCCCATCGACCAGCACCACCG	mNR2F1(-)H	GACAGGTAGCAGTGGCCATTGAGAG	177	2607
mNR2F2(+)C	GAAGATGCAAGCGGTTTGGGAC	mNR2F2(-)D	GGCAAACTGCCCGTGGGT	100	7908
mSox9(+)C	AGTACCCGCATCTGCACAAC	mSox9(-)D	TACTTGTAATCGGGGTGGTCT	145	938
Controls					
mSuz12(+)A	CGGCCACAAGAAATGGAAGTAGATA	mSuz12(-)B	TGCTGCATTTCTCGGAGCTT	335	2753
m36B4(+)A	AGAACAACCCAGCTCTGGAGAAA	m36B4(-)B	ACACCCTCCAGAAAGCGAGAGT	448	629
Genotyping					
mRARαE34(+)	TGGCTCAAACCACTCCATCGAGA	mRARαE6(-)	CCTGGTGCGCTTTGCGAACC	425	n/a
mRARβE3a(+)	GCAGCACCGGCATACTGCTC	mRARβE4(-)	CACTGACGCCATAGTGGTA	155	26450
mRARγ2A(+)	tttcaattgCCatgTACGACTGCATGGAATCGT	mRARγE7(-)	TTGCTGACCTTGGTGATGAGTT	551	6031

Table S2: ChIP primers

**Forward**	Sense primer (5'-3')	**Reverse**	Antisense primer (5'-3')	**Product**	**Position**
mNR2F1-p(+)M	TGCCGCCTGTGCCATTTCTGAT	mNR2F1-p(-)P	CAGCGAGCGAGCTCCCTTCTCT	73	-70;+3
mNR2F1-E(+)A	GTCTTCTCGTTCGTTCGTTTGCTCTT	mNR2F1-E(-)B	CCTCATATTGTTGTGGGGCGGCT	277	-850;-773
mHoxa5-p(+)G	GCCATAATGGGCTGTAACCTCA	mHoxa5-p(-)H	ACCCGTTGCCGCCGTTCAGT	120	-160;-140
mHoxa5-R(+)A	CCTGAAGCTCAGTGCTGTGTATCT	mHoxa5-R(-)B	CTGTCTGGGCAGATGACTAAGAG	112	+7487;+7599
