# Supplementary material for: Polycomb recruitment attenuates retinoic acid–induced transcription of the bivalent NR2F1 gene
Source: Nucleic Acids Res. 2013 May 10;41(13):6430–43. doi: 10.1093/nar/gkt367 (PMC3905905; doi:10.1093/nar/gkt367)
Supplement: Supplementary Data [file supp_gkt367_Laursen_Supp_FINAL.pdf]

SUPPLEMENTARY INFORMATION

ABBREVIATIONS

cDNA:	complementary DNA
Coup-TF:	Chicken Ovalbumin Upstream Promoter-Transcription-Factor
ES:	Embryonic Stem
gDNA:	genomic DNA
H3K27me3:	Trimethylated Histone 3 Lysine 27
H3K4me3:	Trimethylated Histone 3 Lysine 4
IEE:	Inducible Enhancer Element
NR2F1:	Nuclear Receptor 2F1
PCR:	Polymerase Chain Reaction
PRC:	Polycomb Repressive Complex
RA:	all-trans Retinoic Acid
RAR:	Retinoic Acid Receptor
RARE:	Retinoic Acid Responsive Element
RefSeq:	NCBI Reference Sequence
RT:	Reverse Transcription
RXR:	Retinoid X Receptor
shRNA:	short hair-pin RNA
TSS:	Transcriptional Start Site
WT:	Wild-Type

FIGURE LEGENDS

Figure S1. Genotypic validation of F9 RAR knockout cell lines.

Transcript levels were assessed in triplicate samples after 0, 8 and 24 hours treatment with RA. The RAR isoform and PCR band sizes are indicated to the left of the gel. Note the *RARβ*$_2$ is itself induced by RA. The truncated transcripts detected in RARα and RARγ knockout cell lines, respectively, represent nonsense coding mRNAs resulting from the genomic manipulations. The three F9 RAR knockout cell lines were all generated by former lab members (Boylan et al., 1993; Boylan et al., 1995; Faria et al., 1999).

Figure S2. CpG methylation of *Nr2F1* RARE and promoter regions. The CpG methylation is scarce at the enhancer and at the promoter regions of *Nr2F1* both in the absence (blue) and in the presence (red) of RA. Each horizontal line represents the methylation status of an independent allele. The numbers below the figures indicate the CpG position relative to the P_{RefSeq} transcriptional start site (+1).

Figure S3. Co-IP of PRC2 core components Ezh2 and Ezh1. Suz12 interacting proteins were immunoprecipitateded using a Suz12 directed antibody and detected by Western blot analysis. Ezh2 but not Ezh1 associated with Suz12. Note that the Ezh1 bands in the Suz12 IP had intensities similar to those in the IgG negative control (background levels).

Figure S4. The Effect of RA on RARγ, RXRα, and PolII association with *Nr2F1, Nr2F2, Sox9, Hoxa5,* and *Cyp26a1.* RARγ, RXRα, and PolII association were characterized using publically available genome wide chromatin immunoprecipitation assays coupled with next generation DNA sequencing datasets (ChIP-seq). (A) *Nr2F1*, (B) *Nr2F2,* (C) *Sox9*, (D) *Hoxa5*, and (E) *Cyp26a1* genomic loci in mouse F9 stem cells. ChIP-seq for untreated (0 h RA) and RA treated F9 cells (6 h and 48 h RA) are shown for RARγ (purple), RXRα (green), and RNA polII (blue). Each read is aligned to the corresponding genomic location, thereby visualizing specific chromatin association as density of reads. RefSeq intron and exon locations are specified in blue at the bottom of each alignment. Chromosome coordinates and scale bars are located at the top of each alignment.

Figure S5. The Epigenetic signatures of *Nr2F1* and *Hoxa5.* Histone modifications and PRC component association were characterized using publically available genome wide chromatin immunoprecipitation assays coupled with next generation DNA sequencing datasets (ChIP-seq). (A) *Nr2F1*, (B) *Nr2F2,* (C) *Hoxa5* and (D) *Cyp26a1* genes in mouse embryonic stem cells. Accession numbers for datasets used were for ChIPseq: GSE30538 (F9 RARγ, F9 RXRα, F9 Pol II); GSE12241 (ES H3K4me3, ES H3K27me3, ES RNA pol II); GSE11172 (ES H3K4me1, ES H3K4me2); GSE13084 (PRC proteins, ES Suz12, ES EZH2, ES Ring1B); GSE24165 (ES H3K27ac); GSE465889 (Jarid2). RNAseq: mRNA expression analysis of RA differentiated mouse ES cells GSM566812. DNA methylation and hydroxymethylation state: GSE28682.

FIGURES

Laursen_FigS1

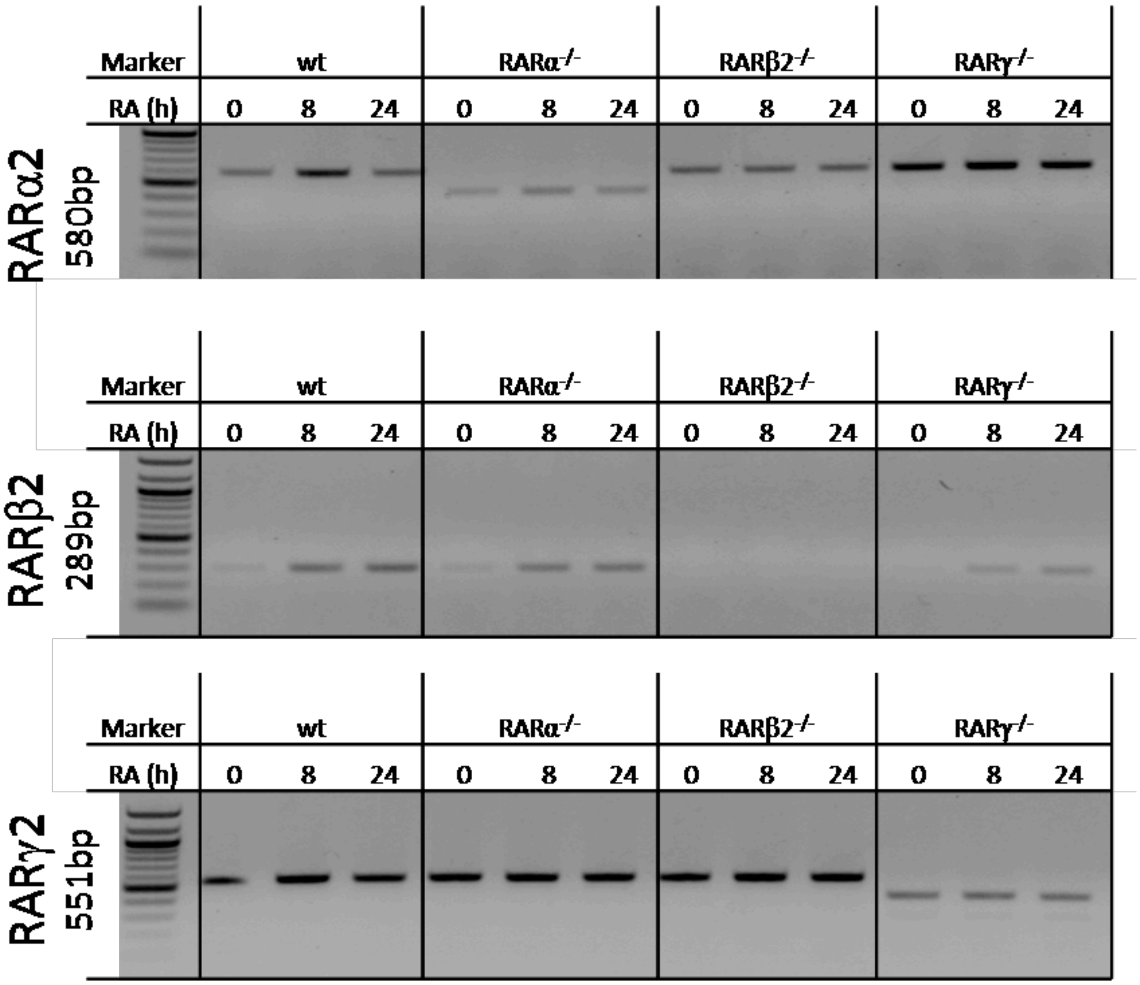

Laursen_FigS2

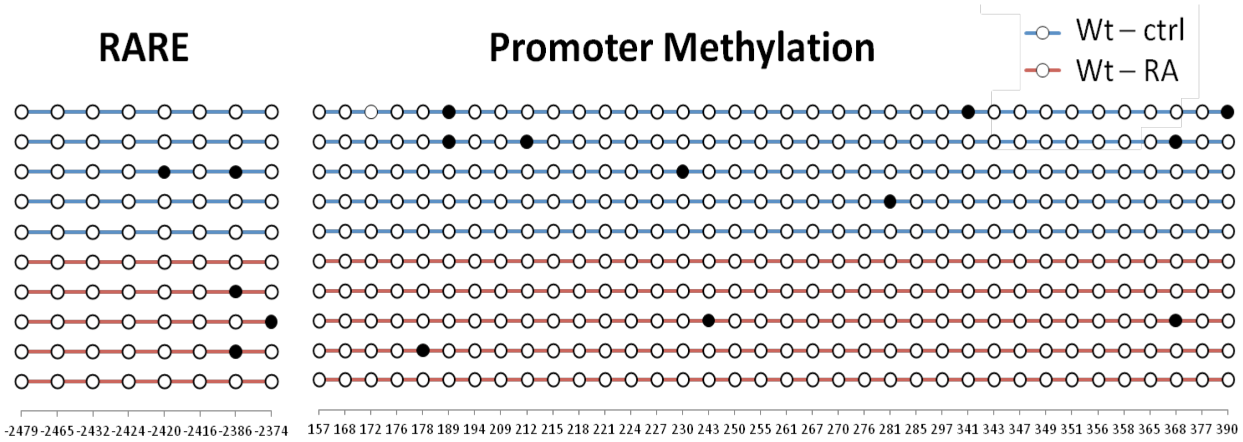

Laursen_FigS3

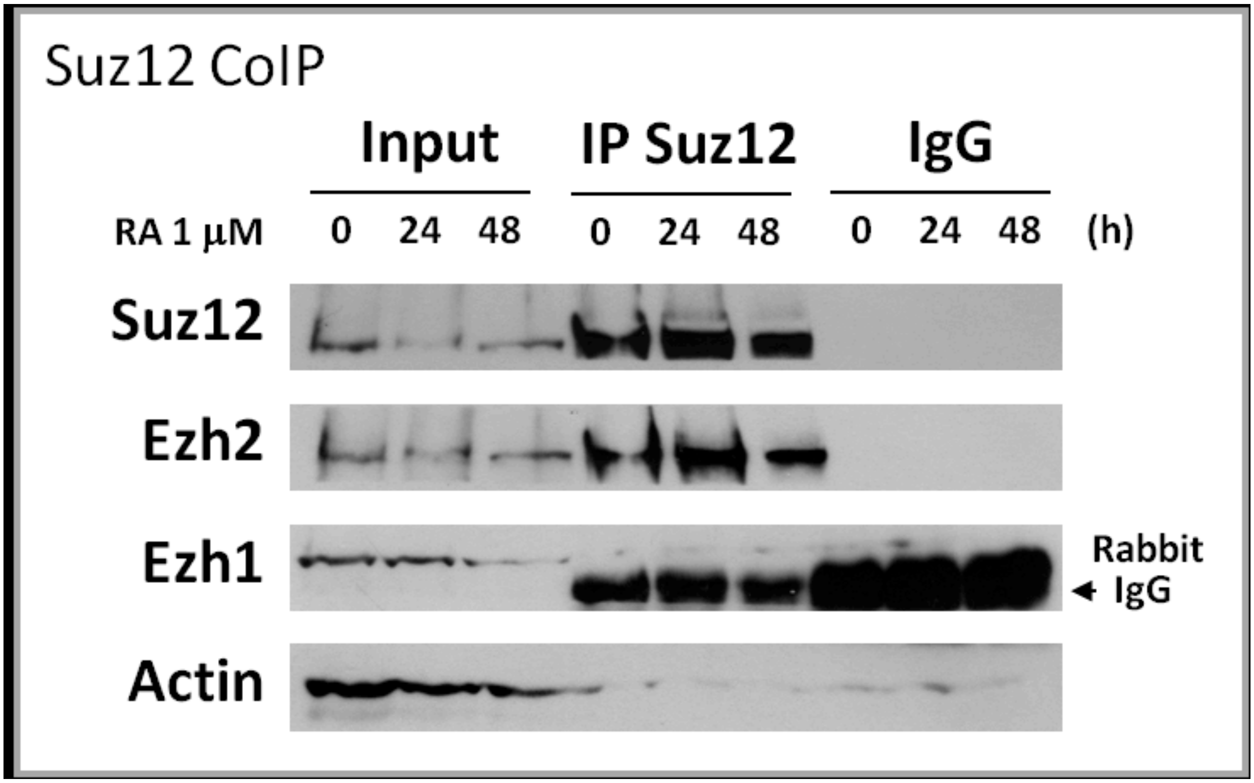

Laursen_FigS4

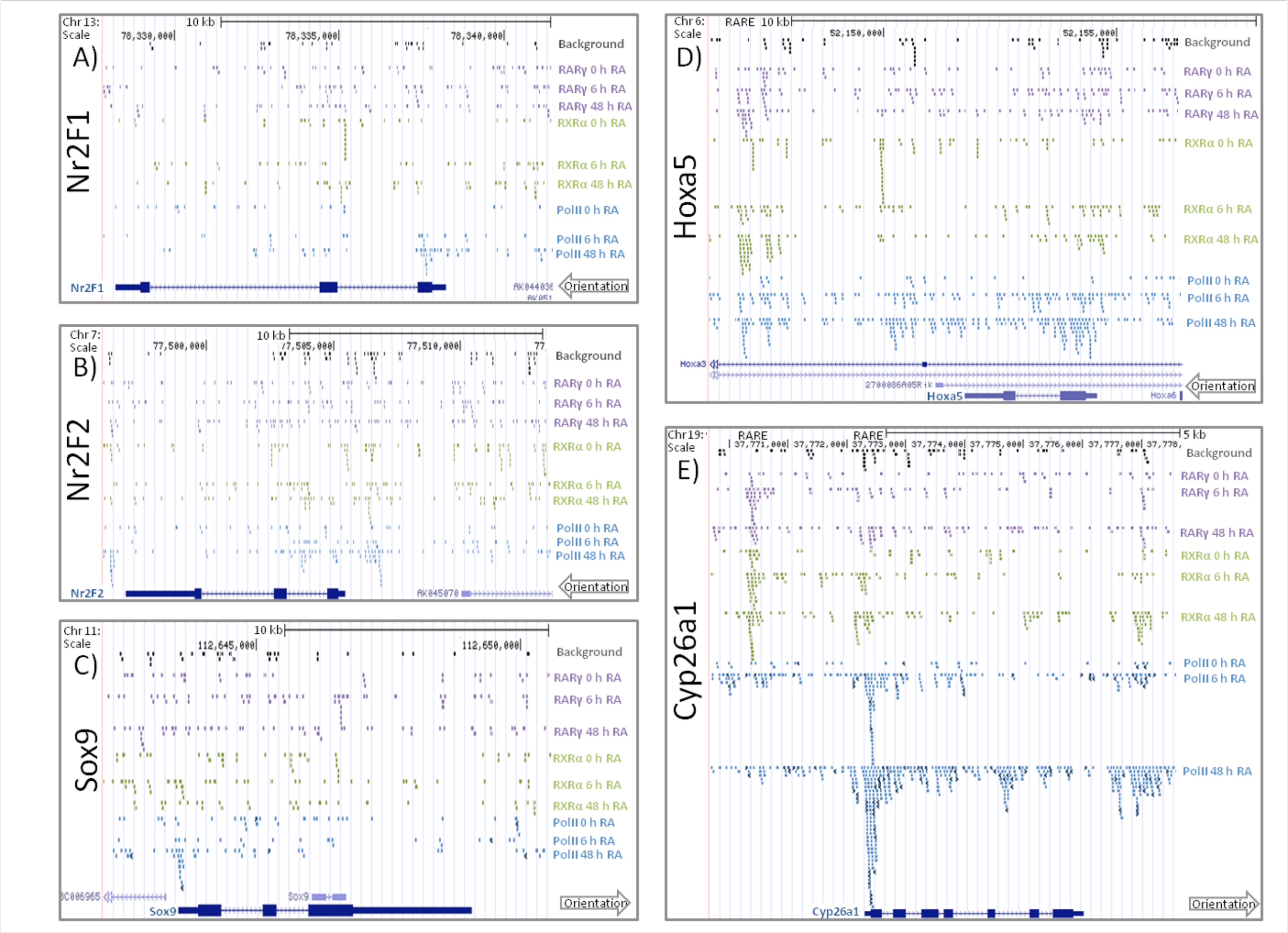

Laursen_FigS5

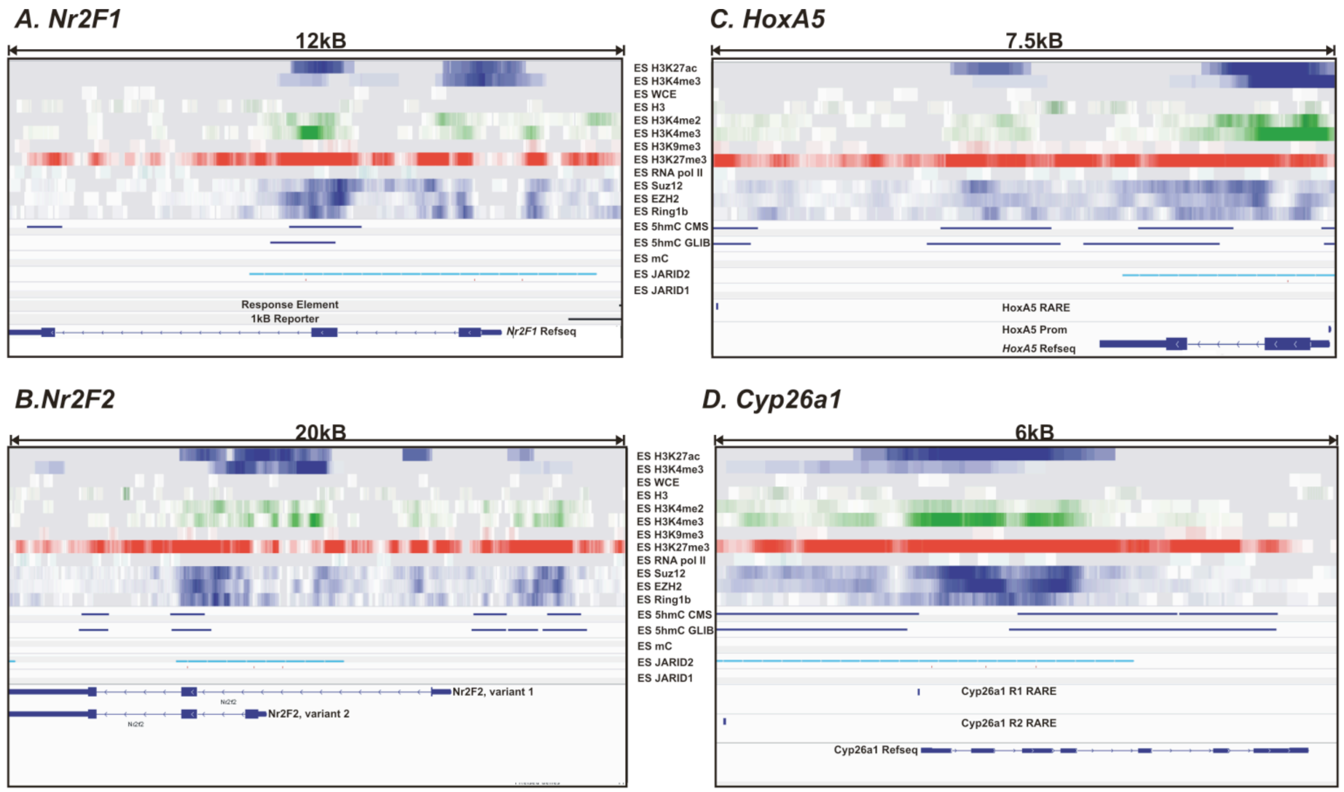

Table 1: Gene Expression Primers

**Forward**	Sense primer (5'-3')	**Reverse**	Antisense primer (5'-3')	**Product**	(bp)
Induced	by RA			cDNA	gDNA
mBMP2(+)A	CTTAGACGGACTGCGGTCTCCTA	mBMP2(-)B	ACGTCTTCCGAAGGCCGGGACA	171	1238
mCyp26a1(+)A	GAAACATTGCAGATGGTGCTTCAG	mCyp26a1(-)B	CGGCTGAAGGCCTGCATAATCAC	272	728
mCyp26b1(+)A	TGGACTGTGTCATCAAGGAGGT	mCyp26b1(-)B	GTCGTGAGTGTCTCGGATGCTA	143	492
mHoxa1(+)E	TAACTCCTTATCCCCTCTCCAC	mHoxa1(-)D	ACCCACGTAGCCGTACTCTCCA	151	628
mHoxa5(+)C	CCCCTGGATGCGCAAGCTGCACATT	mHoxa5(-)F	TTCTCCAGCTCCAGGGTCTGGTAGCGA	105	1062
mMeis1(+)A	CATGATAGACCAGTCCAACC	mMeis1(-)D	GGCTACATACTCCCCTGGCATACT	243	3827
mNR2F1(+)G	CTGTCCCATCGACCAGCACCACCG	mNR2F1(-)H	GACAGGTAGCAGTGGCCATTGAGAG	177	2607
mNR2F2(+)C	GAAGATGCAAGCGGTTTGGGAC	mNR2F2(-)D	GGCAAACTGCCCGTGGGT	100	7908
mSox9(+)C	AGTACCCGCATCTGCACAAC	mSox9(-)D	TACTTGTAATCGGGGTGGTCT	145	938
mSuz12(+)A	CGGCCACAAGAAATGGAAGTAGATA	mSuz12(-)B	TGCTGCATTTCTCGGAGCTT	335	2753
m36B4(+)A	AGAACAACCCAGCTCTGGAGAAA	m36B4(-)B	ACACCCTCCAGAAAGCGAGAGT	448	629
Genotyping					
mRARαE34(+)	TGGCTCAAACCACTCCATCGAGA	mRARαE6(-)	CCTGGTGCGCTTTGCGAACC	425	n/a
mRARβE3a(+)	GCAGCACCGGCATACTGCTC	mRARβE4(-)	CACTGACGCCATAGTGGTA	155	26450
mRARγ2A(+)	tttcaattgCCatgTACGACTGCATGGAATCGT	mRARγE7(-)	TTGCTGACCTTGGTGATGAGTT	551	6031

Table 2: ChIP primers

**Forward**	Sense primer (5'-3')	**Reverse**	Antisense primer (5'-3')	**Product**	**Position**
mNR2F1-p(+)M	TGCCGCCTGTGCCATTTCTGAT	mNR2F1-p(-)P	CAGCGAGCGAGCTCCCTTCTCT	73	-70;+3
mNR2F1-E(+)A	GTCTTCTCGTTCGTTCGTTTGCTCTT	mNR2F1-E(-)B	CCTCATATTGTTGTGGGGCGGCT	277	-850;-773
mHoxa5-p(+)G	GCCATAATGGGCTGTAACCTCA	mHoxa5-p(-)H	ACCCGTTGCCGCCGTTCAGT	120	-160;-140
mHoxa5-R(+)A	CCTGAAGCTCAGTGCTGTGTATCT	mHoxa5-R(-)B	CTGTCTGGGCAGATGACTAAGAG	112	+7487;+7599
